# Red maple (*Acer rubrum* L.) trees demonstrate acclimation to urban conditions in deciduous forests embedded in cities

**DOI:** 10.1371/journal.pone.0236313

**Published:** 2020-07-24

**Authors:** Covel R. McDermot, Rakesh Minocha, Vince D’Amico, Stephanie Long, Tara L. E. Trammell

**Affiliations:** 1 Department of Plant and Soil Sciences, University of Delaware, Newark, DE, United States of America; 2 USDA Forest Service, Northern Research Station, Durham, NH, United States of America; 3 USDA Forest Service, Northern Research Station, Newark, DE, United States of America; Eidgenossische Forschungsanstalt fur Wald Schnee und Landschaft Institut fur Schnee- und Lawinenforschung, SWITZERLAND

## Abstract

The impacts of urbanization, such as urban heat island (UHI) and nutrient loads, can influence tree function through altered physiology and metabolism and stress response, which has implications for urban forest health in cities across the world. Our goal was to compare growth-stimulating and stress-mitigating acclimation patterns of red maple (*Acer rubrum*) trees in deciduous forests embedded in a small (Newark, DE, US) and a large (Philadelphia, PA, US) city. The study was conducted in a long-term urban forest network on seventy-nine mature red maple trees spanning ten forests across Newark and Philadelphia. We hypothesized that red maples in Philadelphia forests compared to Newark forests will be healthier and more acclimated to warmer temperatures, elevated CO_2_ concentrations and reactive nitrogen (N_r_) deposition, and higher nutrient/heavy metal loads. Therefore, these red maples will have higher foliar pigments, nutrients, and stress-indicating elements, enriched δ^15^N isotopes and increased free polyamines and amino acids to support a growth-stimulating and stress-induced response to urbanization. Our results indicate red maples are potentially growth-stimulated and stress-acclimated in Philadelphia forests experiencing a greater magnitude of urban intensity. Red maples in Philadelphia forests contained higher concentrations of foliar chlorophyll, %N, δ^15^N, and nutrients than those in Newark forests. Similarly, lower foliar magnesium and manganese, and higher foliar zinc, cadmium, lead, and aluminum reflected the difference in soil biogeochemistry in Philadelphia forests. Accumulation patterns of foliar free amino acids, polyamines, phosphorous, and potassium ions in red maples in Philadelphia forests shows a reallocation in cellular metabolism and nutrient uptake pathways responsible for physiological acclimation. Our results suggest the approach used here can serve as a model for investigating ‘plant physiology’ and the use of urban trees as a biomonitor of the impacts of ‘urban pollution’ on urban forests. The results suggest that cellular oxidative stress in trees caused by pollutant uptake is mitigated by the accumulation of free amino acids, polyamines, and nutrients in a larger city. Our study provides a framework for determining whether trees respond to complex urban environments through stress memory and/or acclimation.

## Introduction

The magnitude of urbanization across cities alters abiotic conditions that can influence tree physiology and metabolism, which has global implications for urban forest health [1–3] as cities across the world continue to experience greater human impacts and expansion [4]. Cities are an ideal ‘natural experiment’ for assessing the cumulative impacts of current global environmental changes [5–7]. Forest trees are subjected to increased atmospheric reactive nitrogen deposition inputs [8–10], greater CO_2_ concentrations [11,12], and higher temperatures [13,14] that have the potential to stimulate growth. However, increased soil heavy metal concentrations [15,16] and reduced soil moisture [17] due to UHI have the potential to stress trees in urban forests. Krämer [18] reported many stress-inducing metals have no reported threshold deficiency levels, yet critical toxicity levels have been identified. Foliar physio-biochemical response patterns to above- and below-ground conditions in urban landscapes can provide valuable insights into the regulation of tree growth [3,19–21].

Trees in cities experience a wide range of environmental pressures that create both growth stimulating and stress inducing conditions. For example, UHI can stimulate plant growth [13,17,21] or alternatively lead to drought stress and dampened plant growth [22,23]. In addition, elevated atmospheric N_r_ deposition can stimulate plant growth or induce nutrient deficiencies depending upon the initial N status (land use history) of a site [8,24–27]. Similarly, storm run-off, wet and dry deposition, and environmental legacies can exacerbate soil nutrient and toxin levels that can either negatively or positively impact plant growth [16,28,29].

Within individual tree species and genotypes, foliar pigments and biochemical traits may be a summation of the response to long-term and current above- and below-ground site conditions that dictate tolerance limits for elements present in the environment [30–33]. Therefore, above- and below-ground conditions in an urban environment can simultaneously impact the physiology and biochemistry of trees that might create a tight coupling of patterns in soil and foliar biochemistry. Plant species that grow in adverse environmental conditions can exhibit biochemical and physiological change brought on by abiotic stressors [34–39]. The physiological relationship between abiotic stress and nitrogen-utilizing amino acids and polyamines, as well as some exchangeable ions in plants, has been explored and proposed as a suite of potential physio-biochemical indicators of persistent environmental stresses [40–42]. Observed increases in tolerance to abiotic stress when cellular polyamines, amino acids, and nutrients are elevated is indicative of a protective role (antioxidation, osmoprotection, intracellular signaling and metal-chelation) to cellular organelles such as chloroplasts and mitochondria. This results in the establishment of new cellular reactive oxygen/nitrogen species (ROS, RNS) homeostasis in trees in which phenotypic symptoms of stress are not yet evident [36,41,43,44]. Thus, foliar pigments, amino acids, polyamines, and nutrient ions can act as a suite of combined physiological biomarkers of changes in soil biogeochemistry and atmospheric conditions in cities.

Many studies have focused on drivers of plant physiological and metabolic traits (net photosynthesis rate, chlorophyll content, C/N ratio, polyamine and amino acid content, and nutrients [25,45,46] in contrasting environments and experimental forests. However, there is difficulty in conducting long-term manipulated experiments *in situ* that mimic global change impacts (urbanization) on physio-biochemical plant traits [5]. Cities are experimentally underutilized and can serve as a surrogate for such purpose. The magnitude of urban intensity varies with city size and is unique to each city. Thus, different above- and below-ground controls related to city size (urban heat island intensity) can influence plant physiology in different pathways, and trees in urban forests can be biomonitors of these variable environmental conditions and impacts. Research that exploits this fact with an interdisciplinary approach will provide baseline scientific data on ‘urban tree physiology’ and will develop this field further. Previous work on tree response to urban conditions utilized a gradient approach of urbanization intensity from one large city [10,12,29,46–49], yet the majority of cities in the U.S. have low-intensity land cover and more open-area developed spaces. Thus, to build collective understanding of how multiple environmental changes influence tree physiology, we need research that focuses on tree response in multiple size cities with differing magnitudes of urban intensities (metro-scale impacts). Furthermore, a comparison of tree physiology and biochemistry in cities with different sizes can help isolate the controls (metro-scale versus site-scale) on tree response to urban environmental conditions.

To understand the impacts of different magnitudes of urbanization intensity on the physiology and stress mitigating-acclimating response (i.e., effects on pigments and free metabolites) in cities, we investigated mature native red maples in small deciduous forests embedded in two cities (i.e., Newark, DE and Philadelphia, PA). These cities differ in total population, developed land area, and impervious surface cover, and provide a natural experiment for discerning urban impacts on tree physiology and biochemistry response to urbanization. This research utilized the long-term urban forest ecology network, FRAME (FoRests Among Managed Ecosystems), to study how red maples respond to urbanization in a small city (Newark, DE) and a large city (Philadelphia, PA). We measured the concentrations of foliar pigments (chlorophyll and carotenoids), foliar nitrogen (%N and δ^15^N isotope), nutrient elements (Ca, Mg, P, K, S, Fe, Mn), stress-inducing elements (Ni, Zn, Al, Na, Cd, Cu, Co, Cr, Se, Pb), stress mitigating and signaling free polyamines (putrescine, spermidine and spermine), and free amino acids (arginine [Arg], threonine [Thr], proline [Pro], *gamma*-amino butyric acid [GABA], ornithine [Orn], glycine [Gly], glutamine [Gln], and glutamic acid [Glu]) in red maple trees. We also measured multiple soil attributes; sub-canopy nutrient elements, stress-inducing elements, bulk density, organic matter, and pH. We posed the following questions: 1) Do red maple foliar chlorophyll, carotenoids, chlorophyll/carotenoid ratio, nitrogen, nutrient, and stress-inducing metal concentrations in urban forests differ with city size; and 2) Do observed differences in foliar and soil chemistry between cities indicate differing metabolic responses? We hypothesized that red maples growing in forests in Philadelphia as compared to those in Newark will have higher foliar pigments, nutrients, and stress-inducing elements, and more enriched δ^15^N isotope due to greater nutrient and metal inputs, urban heat island, and elevated atmospheric CO_2_ that create stronger above- and below-ground controls with respect to city size leading to stress acclimation. We also hypothesized that higher foliar polyamines, some amino acids, and nutrients in trees in Philadelphia forests relative to Newark forests would indicate greater stress tolerance to a greater magnitude of urban intensity impacts (UHI, N_r_ deposition inputs, anthropogenic CO_2_, nutrient/toxic metal loads) of a larger city. The results from this study provide valuable insight into how above- and below-ground conditions in cities drive differing patterns in tree physiological response based on the magnitude of the (city size as a proxy for) urbanization intensity.

## Materials and methods

### Study species and geographic region

**Fig 1:**
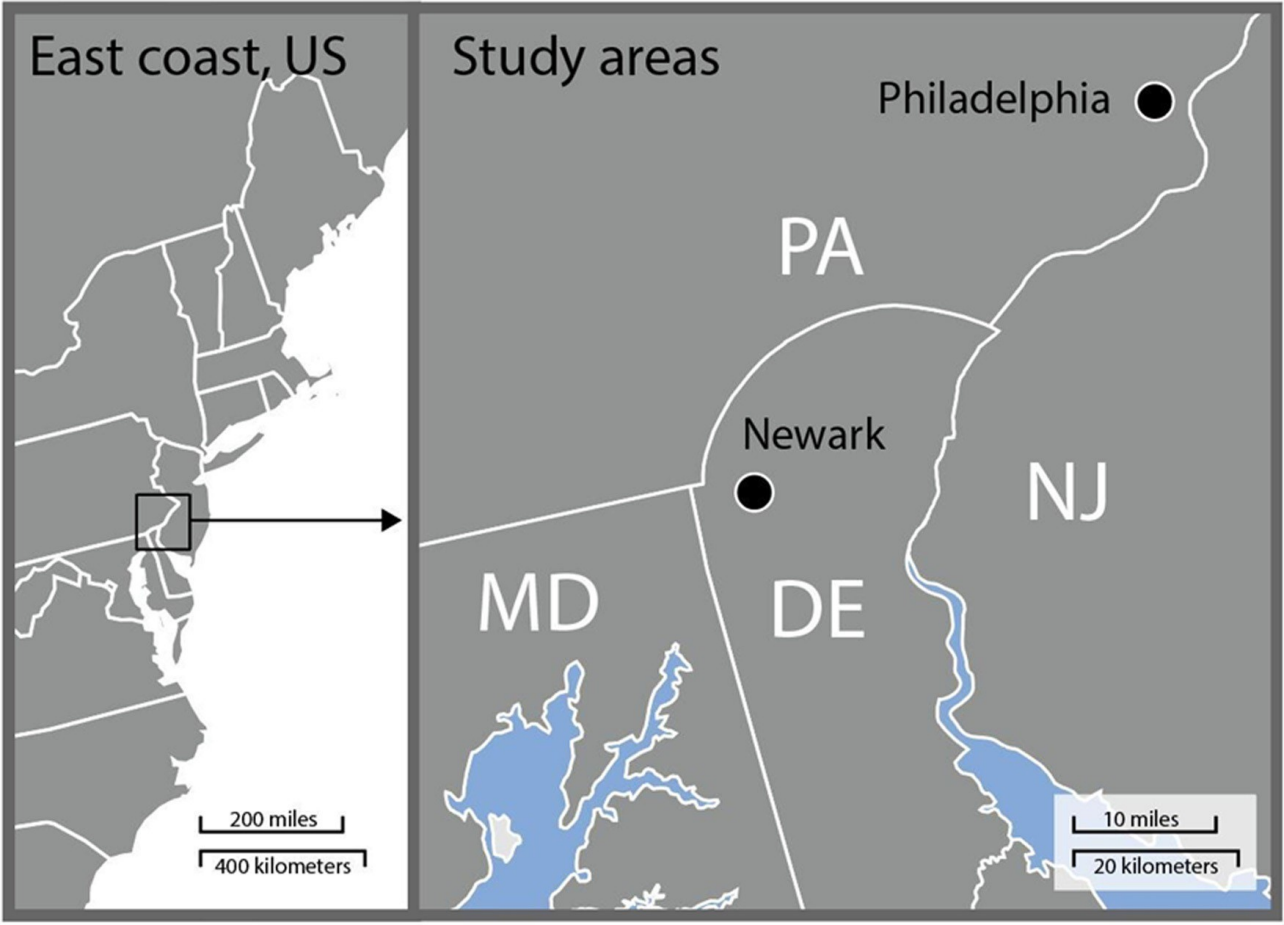
Map of the study area. Map shows the east coast of the U.S. and the location of Newark, DE and Philadelphia, PA. A total of 79 red maple trees from ten forests were sampled in August 2017.

In the present study, we focused on mature red maples (*Acer rubrum* L.) in ten forests in the long-term urban forest ecology network, FRAME (**F**o**R**est **A**mong **M**anaged **E**cosystems); five in Newark, DE and five in Philadelphia, PA, U.S (Fig 1). In 2009, researchers at the USDA Forest Service and University of Delaware selected forest sites located on public lands across the Coastal Plain and Piedmont region of the Mid-Atlantic for long-term establishment and research. The FRAME traverses an urban gradient extending across Newark, DE and Philadelphia, PA into nearby rural areas. Newark has a population of 31,454 and a mean density of 1,403 people km^-2^. Philadelphia has a population of 1,526,006 people and density of 4,405 people km^-2^ [50]. In both cities, the forest canopy is dominated by red maple (*Acer rubrum*), tulip poplar (*Liriodendron tulipifera*), American beech (*Fagus grandifolia*), black gum (*Nyssa sylvatica*), sweetgum (*Liquidambar styraciflua*), and red oak (*Quercus rubra*). The forest understory is dominated by a mix of non-native and native species including multiflora rose (*Rosa multiflora*), spicebush (*Lindera benzoin*), greenbrier (*Smilax rotundifolia*), Japanese honeysuckle (*Lonicera japonica*), southern arrowwood (*Viburnum dentatum*), autumn olive (*Elaegnus umbellata*), and sweet pepperbush (*Clethra alnifolia*).

### Experimental design, tree selection, and sample collection

For this study, we selected five small forests experiencing high levels of urbanization in Newark, DE (forest size: 4.0–16.3 ha) and in Philadelphia, PA (forest size: 3.5–7.0 ha); therefore, focusing this study on the impact of large-scale urbanization pressures associated with city size on tree physiology and metabolism. The urban context surrounding forests in Newark and Philadelphia was similar in land use/land cover, impervious surface area, and population density (see Trammell et al. 2020 for details on landscape metrics and spatial analyses). We randomly selected 5 or 10 red maple trees in five forests in Newark (n = 39) and Philadelphia (n = 40) depending upon availability and proximity to each other. Seventy-nine mature, native red maple trees (diameter at breast height [dbh]: 7.6–23 cm) were randomly sampled at mid- to upper-canopy in August 2017. Red maples in Newark (dbh: 13.7 ± 0.5 cm) and Philadelphia (dbh: 12.2 ± 0.5 cm) forests did not differ significantly in mean dbh (*p* = 0.06) and were assumed to be similar in age. The trees were located at least 10 m from the forest edge, and many trees were near a creek and/or close to a trail. The trees sampled experienced sub-canopy shade as they were not tall enough to reach the upper forest canopy. No specific permissions were required for locations/activities as these forest fragments because they are part of our FRAME network. The FRAME is open to graduate and undergraduate students and postdoctoral scholars to carry out long- and short-term experimental studies that do not involve endangered species.

Leaf sampling was conducted at mid-upper canopy using a pole pruner, a slingshot, or by climbing the tree in August 2017. Light-exposed leaf samples were stored on ice during collection in the field and transport to the laboratory. Foliar samples were stored at -20°C for pigment analysis, and a portion was oven dried at 55°C for analyses of nutrients, stress-inducing metals, %N, and natural abundance δ^15^N isotope. Leaf disks from three to five fresh clean leaves were collected in the field with a paper puncher to obtain ~100 mg of tissue. The sample was placed into a pre-weighed microfuge tube and 1 ml of 5% perchloric acid (PCA; stops biochemical reactions instantly) was added. Samples were stored and transported on ice and were placed at -20°C until analyses of polyamines, amino acids, and exchangeable ions.

Under each red maple tree, a composite of four soil cores (0–10 cm) were collected at the mid-point between the tree trunk and canopy edge for elemental analysis. One intact soil core (diameter = 5 cm, depth = 15 cm) was also collected under each tree for bulk density (BD), organic matter (SOM) and pH measurements. Soil samples measured for BD measurements were oven dried at 105°C.

### Foliar %N and natural abundance δ^15^N isotope measurement

All leaves were oven-dried at 55°C for 48 h and subsamples (~ 3500 μg) were ground to a fine powder using a Retsch Ball Mixer Mill (MM200, Haan, Germany). Nitrogen (N) concentrations (%) and δ^15^N values (‰) were measured using an Elemental Combustion System (ECS) 4010 CHNSO analyzer, Costech Analytical (Costech Valencia, CA, USA) connected to a Thermo Delta V spectrometer (Thermo, Bremen, Germany) interfaced with an elemental analyzer. Two USGS standards (external) and one secondary standard (acetanilide, internal) were used, with precision less than 0.02‰ [51]. The natural abundance stable isotope values were expressed relative to the international standard (atmospheric N_2_) in the conventional δ-notation [51]:

$$\delta^{15}\text{N}=\left[\left({}^{15}{\text{N}}_{\text{sample}}{/}^{14}{\text{N}}_{\text{sample}}\right)/\left({}^{15}{\text{N}}_{\text{standard}}{/}^{14}{\text{N}}_{\text{standard}}\right)\;–\;1\right]\;*\;1000‰$$ (1)

### Foliar chlorophyll and carotenoid concentration

Freshly frozen leaf samples were brought to room temperature and a paper puncher used to remove three discs (~ 8.5–11.5 mg). One ml of Dimethylformamide (DMF) was added to the leaf discs and shaken for 24 h in the dark, then transferred to a refrigerator at 4°C for 48 h for complete pigment extraction. The extracts were filtered and further diluted with DMF to attain absorbance values below 1 absorbance in accordance with Beer’s Law. Absorbance were measured in triplicate at wavelengths of 480, 647 and 664 nm using a spectrophotometer (Thermo Evolution 60S UV-Visible, Waltham, MA, USA). Total chlorophyll (*a* + *b*) and carotenoid concentrations were calculated according to equations by Porra et al. [52] and Minocha et al. [53]:

[Chlorophylla]equation=(12×A664)−(3.11×A647)×dilutionfactor (2)

[Chlorophyllb]equation=(20.78×A647)−(4.88×A664)×dilutionfactor (3)

[Totalchlorophyll(a+b)]equations=[Chlorophylla]+[Chlorophyllb] (4)

[Totalcarotenoids]equation=(1000A480−1.12Ca−34.07Cb)/245×dilutionfactor (5)

### Foliar polyamine, amino acid, and exchangeable ion measurement

To extract 5% PCA soluble polyamines and amino acids, samples were thawed and frozen (-20°C) three times and then centrifuged at 13 000 × *g* for 10 min [54]. The supernatants were dansylated for the simultaneous analysis of polyamines and amino acids according to the procedure described in Minocha and Long [55] with minor modifications described here. In short, the changes include a 30 min incubation time, termination of the dansylation process with 45 μl of glacial acetic acid, and final volume of 2 ml. Following this protocol, the separation of Arginine (Arg) and Threonine (Thr) was not always complete. For quantitation a combined calibration curve of Arg+Thr was used; when both AAs are present in equal concentration, the area of Arg is a much larger percentage of the combined peak area. Detection limits, recovery, linear ranges, precision and error for this method are all described in [55]. Exchangeable inorganic ions (soluble in 5% PCA) were quantified from the same PCA extracts using a simultaneous axial inductively coupled plasma optical emission spectrophotometer (ICP-OES) (Vista CCD, Varian Inc., Palo Alto, CA, USA) and Vista Pro software (Version 4.0), following 100 × dilutions of the PCA extracts with deionized water [56]. ICP analysis was done in accordance with EPA SW-846 compendium, method 6010.

### Foliar and soil nutrient and heavy/toxic metal concentrations

Leaf samples (~ 0.5 g) were oven-dried at 55°C for 48 h and ground to fine powder prior to digestion with a CEM MARs5 microwave digestion system (CEM, Matthews, NC, USA) using concentrated nitric acid and 30% hydrogen peroxide. The supernatants were analyzed for the contents of Boron (B), Ca, Mg, K, P, S, Mn and Fe by inductively coupled plasma optical emission spectroscopy (ICAP; 7600 Duo view Inductively Coupled Plasma–Optical Emission Spectrometer [ICP-OES], Thermo Elemental, Madison, WI, USA) and other elements (Na, Al, Cu, Cr, Co, Ni, Zn, Cd, As, Se and Pb) by Inductively Coupled Plasma Mass Spectrophotometer (ICP-MS; Agilent 7500cx, Wilmington, DE, USA). Helium gas was introduced into the octopole reaction cell for the analysis of Cr, Co, Ni, As and Se [57].

Composite soil samples collected at 0–10 cm depth were dried at 105°C for 48 h, sieved with a 2 mm pore sieve, and ground to fine powder (Retsch MM200, Haan, Germany). Soils (0.5 g) were extracted using the Mehlich 3 soil test extractant protocol [58]. The supernatants were analyzed for the contents of B, P, K, Ca, Mg, Mn, Fe, and S by ICP-OES and other elements (Na, Al, Zn, Cr, Co, Cu, Ni, As, Se, Cd and Pb) using ICP-MS using the same protocol described for foliar samples above.

### Measurements of soil bulk density, organic matter content and pH

Soil samples were oven-dried at 105°C prior to analysis. Intact soil core dried mass to total volume ratios were calculated to determine the soil BD. SOM content was analyzed by the combustion method, loss on ignition (LOI) and carried out at 350°C [59]. Soil pH (1:1; was determined on sub-samples according to Eckert and Sims [60]).

### Statistical analysis

All data analyses were conducted in R (Version 3.3.3 and x64 3.6.1; R Core Team, 2016). Statistical significance is reported at the critical level (α = 0.05). Analyses of differences in foliar chlorophyll (mg g^-1^ FW), carotenoid (mg g^-1^ FW), % N, δ^15^N (‰), amino acids, and polyamines between the cities were analyzed using a one-way analysis of variance (ANOVA) for data that met the assumptions of normality and homoscedasticity, followed by post-hoc Tukey HSD tests. Additionally, the Kruskal-wallis non-parametric analysis of variance was performed when the assumptions of normality and homoscedasticity were not met. Principal component analysis (PCA) was used to determine whether patterns in foliar chemistry (nutrients and stress inducing metals; mg kg^-1^ DW, μg kg^-1^ DW or μmol g^-1^ FW) and metabolism (amino acids and polyamines; nmol g^-1^ FW), and soil chemistry (nutrients and stress inducing metals; mg kg^-1^ DW or μg kg^-1^ DW) were discernible among trees (Version 3.3.3; R Core Team, 2016). PCAs were conducted separately for foliar metabolites and soil chemistry to explore potential differences in foliar pigments, elements, and metabolites in response to soil chemistry.

## Results

### Foliar chlorophyll, carotenoid, N, and δ^15^N

Total foliar chlorophyll concentrations were significantly greater in red maples in Philadelphia forests than in Newark forests (*p* = 0.004; Table 1). Similarly, mean total chlorophyll-*a* (*p* = 0.002) and chlorophyll:carotenoid ratios (*p* = 0.044) were also significantly higher in foliage of red maples in Philadelphia forests compared to those in Newark forests (Table 1). There were no significant differences in total carotenoid concentration in red maples between Philadelphia and Newark forests (*p* = 0.08). Mean foliar %N and naturally abundant δ^15^N were both significantly higher in Philadelphia forests compared to Newark forests (*p* < 0.001 and *p* < 0.02, respectively; Table 1).

**Table 1:** Total chlorophyll, chlorophyll-*a*, carotenoids, chlorophyll:carotenoid (Chl:Car) ratio, N content, and natural abundance δ^15^N for red maple trees in five forests each in Newark, DE (n = 39) and Philadelphia, PA (n = 38).

Forest	Chlorophyll	Chlorophyll-*a*	Carotenoids	Chl:Car	N (%)	δ ^15^N (‰)
Newark						
FO	3.40 ± 0.18	2.40 ± 0.13	1.22 ± 0.05	2.79 ±0.04	1.47 ± 0.07	-2.44 ± 0.4
EW	3.12 ± 0.18	2.20 ± 0.06	1.16 ± 0.06	2.69 ±0.03	1.58 ± 0.05	-10.60 ± 1.2
CW	3.10 ± 0.15	2.16 ± 0.11	1.10 ± 0.05	2.88 ±0.10	1.53 ± 0.03	-11.51 ± 0.7
RH	3.23 ± 0.20	2.03 ± 0.16	1.21 ± 0.06	2.65 ±0.08	1.61 ± 0.11	-8.79 ± 0.8
WF	3.12 ± 0.14	2.20 ± 0.11	1.17 ± 0.51	2.66 ±0.03	1.68 ± 0.05	-11.62 ± 0.3
**Average**	**3.17 ± 0.08**^**a**^	**2.25 ± 0.07**^**a**^	**1.16 ± 0.03**^**n.s.**^	**2.74 ±0.04**^**a**^	**1.57 ± 0.03**^**a**^	**-9.6 ± 0.6**^**a**^
Philadelphia						
PL	3.60 ± 0.40	2.57 ± 0.30	1.30 ± 0.16	2.79 ±0.05	1.71 ± 0.06	-11.68 ± 0.4
SM	3.60 ± 0.22	2.58 ± 0.20	1.24 ± 0.09	2.97 ±0.18	1.79 ± 0.05	-6.87 ± 0.7
WR	3.16 ± 0.12	2.30 ± 0.10	1.15 ± 0.33	2.74 ±0.04	1.58 ± 0.04	-8.59 ± 0.5
PP	3.71 ± 0.22	2.70 ± 0.15	1.35 ± 0.08	2.76 ±0.02	1.52 ± 0.05	-7.24 ± 1.5
CHX	3.72 ± 0.14	2.69 ± 0.10	1.24 ± 0.06	3.03 ±0.11	1.93 ± 0.05	-9.87 ± 0.3
**Average**	**3.53 ± 0.09**^**b**^	**2.54 ±0.07**^**b**^	**1.24 ± 0.03**^**n.s.**^	**2.88 ±0.06**^**b**^	**1.72 ± 0.03**^**b**^	**-8.43 ± 0.4**^**b**^

Forest: FO, Folk; EW, Ecology Woods; CW, Chrysler Woods; RH, Rittenhouse; WF, Webb Farm; PL, Park Line Dr; SM, Smith Memorial; WR, Walton’s Run; PP, Pennypack Dr; CHX, Chamounix.

Letters ‘a’ and ‘b’ represent significant differences between means when α = 0.05.

Bold values represent significantly greater concentrations.

^n.s.^ denotes ‘not significant.’

Concentrations of pigments are in mg g^-1^ FW.

### Total and exchangeable foliar nutrients, and stress-inducing metals

Foliar total concentrations of P, K, and S were significantly higher, but Mg and Mn were significantly lower in red maples in Philadelphia forests compared to red maples in Newark forests (*p* < 0.05; Table 2). Similarly, exchangeable P and K concentrations were also significantly higher, and Mg and Mn were significantly lower in red maples in Philadelphia forests compared to red maples in Newark forests (*p* < 0.05; Table 3). Red maples in Newark forests had significantly greater foliar concentrations of total Ni (*p* = 0.012), Cr (*p* = 0.02), and Co (*p* = 0.006), whereas in Philadelphia forests they had significantly greater foliar total concentrations Pb (*p* = 0.03) and Cd (*p* = 0.04; Table 2). Although foliar total concentrations of Al and Zn in red maples were not significantly different between cities (Table 2), concentrations of exchangeable Al (*p* = 0.04) and Zn (*p* = 0.003) were significantly higher in Philadelphia forests compared to Newark forests (Table 3).

**Table 2:** Concentrations of total foliar elements in red maples from five forests each in Newark, DE (n = 39) and Philadelphia, PA (n = 38).

Total Element	Newark, DE forests (n = 5)	Philadelphia, PA (n = 5)	Unit
Ca	8330 ± 349^n.s.^	7907 ± 332.6^n.s.^	mg kg^-1^ DW
K	6610 ± 211.5^a^	**8234 ± 305**^**b**^	mg kg^-1^ DW
Mg	**2355 ± 120**^**a**^	2089 ± 102^b^	mg kg^-1^ DW
P	1198 ± 45^a^	**1369 ± 63**^**b**^	mg kg^-1^ DW
S	1019 ± 18^a^	**1099 ± 18**^**b**^	mg kg^-1^ DW
Fe	64.5 ± 2^n.s.^	67.8 ± 1.7^n.s.^	mg kg^-1^ DW
Mn	**714 ± 45**^**a**^	425 ± 33^b^	mg kg^-1^ DW
Zn	38 ± 0.12^n.s.^	41 ± 0.12^n.s.^	mg kg^-1^ DW
Cu	8.5 ± 0.4^n.s.^	8.7 ± 0.5^n.s.^	mg kg^-1^ DW
Na	14 ± 3^n.s.^	13 ± 1^n.s.^	mg kg^-1^ DW
Al	32 ± 1^n.s.^	30.9 ± 1^n.s.^	mg kg^-1^ DW
Cr	**14 ± 1.2**^**a**^	9.2 ± 0.8^b^	μg kg^-1^ DW
Co	**3.6 ± 1.1**^**a**^	0.6 ± 0.08^b^	μg kg^-1^ DW
Ni	**25 ± 4.4**^**a**^	14.6 ± 1.2^b^	μg kg^-1^ DW
Cd	2.3 ± 0.3^a^	**3.3 ± 0.4**^**b**^	μg kg^-1^ DW
As	0.55 ± 0.04^n.s.^	0.27 ± 0.03^n.s.^	μg kg^-1^ DW
Se	2.9 ± 0.18^n.s.^	2.8 ± 0.14^n.s.^	μg kg^-1^ DW
Pb	4.9 ± 0.5^a^	**8.2 ± 1**^**b**^	μg kg^-1^ DW

Letters ‘a’ and ‘b’ represent significance at α = 0.05.

Bold values represent significantly greater concentrations.

^n.s.^ denotes ‘not significant.’

**Table 3:** Concentrations of exchangeable foliar elements (μmol g^-1^ FW) in red maples from five forests each in Newark, DE (n = 39) and Philadelphia, PA (n = 38).

Exchangeable Elements	Newark, DE	Philadelphia, PA
K	35.9 ± 1.4^a^	**48.5 ± 3**^**b**^
Mg	**26.8 ± 1.9**^**a**^	23.1 ± 1.4^b^
P	7.7 ± 0.3^a^	**9.3 ± 0.7**^**b**^
Mn	**2.7 ± 0.3**^**a**^	1.6 ± 0.2^b^
Al	0.1 ± 0.01^a^	**0.2 ± 0.02**^**b**^
Zn	0.2 ± 0.01^a^	**0.3 ± 0.04**^**b**^
Ca	44.7 ± 2.3^n.s.^	46.4 ± 3.7^n.s.^
Fe	0.16 ± 0.013^n.s.^	0.2 ± 0.017^n.s.^
S	1.6 ± 0.07^n.s.^	1.7 ± 0.1^n.s.^
Cu	0.1 ± 0.024^n.s.^	0.1 ± 0.033^n.s.^
Ni	0.01 ± 0.002^n.s.^	0.01 ± 0.003^n.s.^
Se	0.003 ± 0.001^n.s.^	0.003 ± 0.001^n.s.^
Co	0.004 ± 0.0005^n.s.^	0.003 ± 0.0004^n.s.^
As	0.006 ± 0.0001^n.s.^	0.01 ± 0.0001^n.s.^
Cd	0.025 ± 0.01^n.s.^	0.03 ± 0.02^n.s.^
Pb	0.003 ± 0.001^n.s.^	0.002 ± 0.001^n.s.^

Letters ‘a’ and ‘b’ represents significant differences between means when α = 0.05.

Bold values represent significantly greater concentrations.

^n.s.^ denotes ‘not significant’.

**Fig 2:**
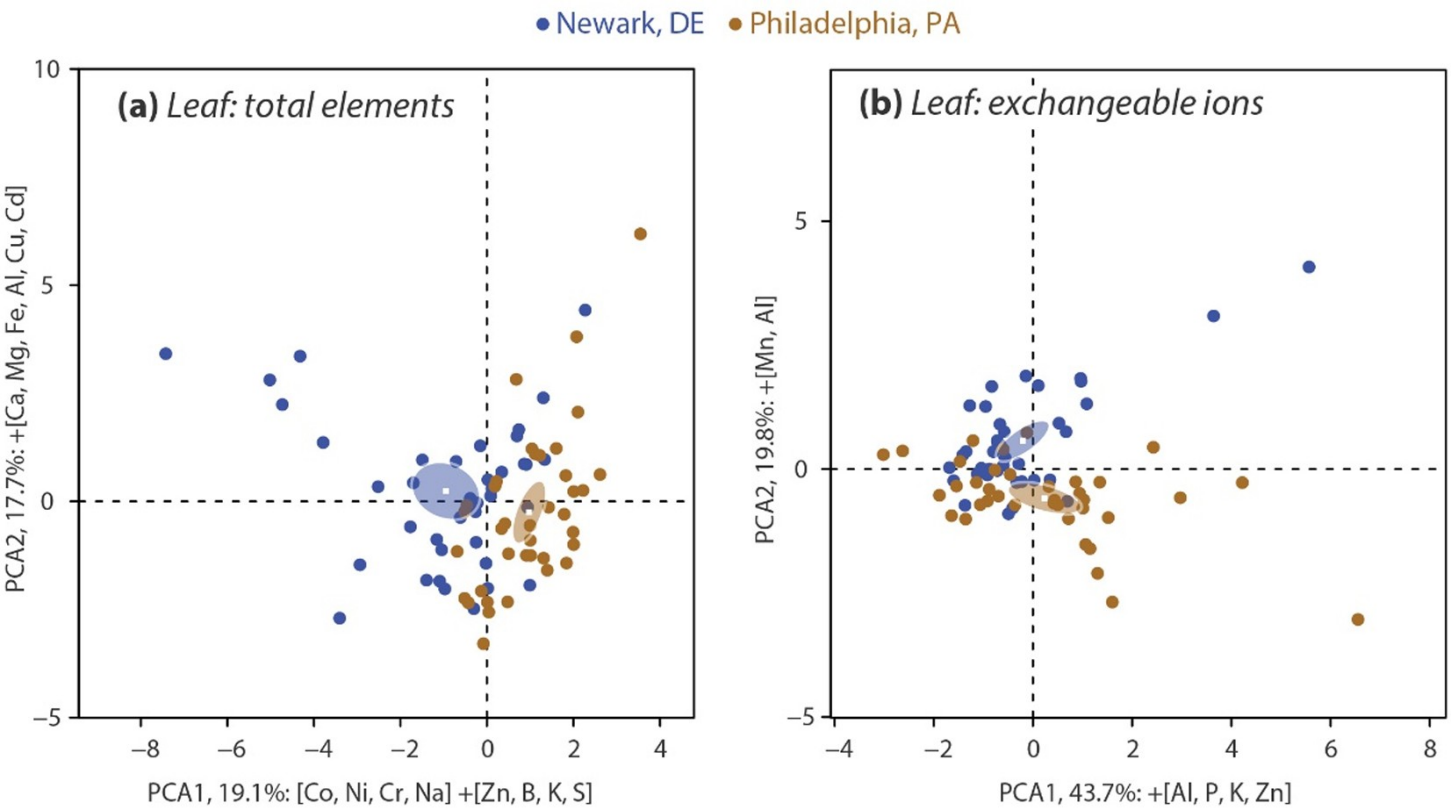
Principal component analysis of foliage of 77 mature native red maples for total (a) and exchangeable (b) elemental composition in ten urban deciduous forests in Newark, DE and Philadelphia, PA. Elements that most strongly loaded on each axis are listed next to their respective axis in order of strongest to weakest. (+) represents values that are positive and strongly loaded on the axis; (-) represents values that are negative and strongly correlated to the axis. The ellipses represent the group mean for each city.

Principle component analysis (PCA) was used to characterize the relationship of foliar elements in red maples in Newark and Philadelphia forests. The first two principle components for the foliar total ion chemistry described 36.8% of the variation among the elements (Fig 2A) which was not very high likely due to substantial variation and/or little separation. PCA1 and PCA2 variables explained 19.1% and 17.7%, respectively, of the variation in correlation in the foliar elements of the red maple trees in both cities (Fig 2A). The variables having the strongest correlation with PCA1 were Co and Ni (negative), and Zn (positive) in the order of strongest to weakest. The variables most significantly correlated on PCA2 were Ca and Mg (in the order of strongest to weakest) and all had strong, positive loadings. As for exchangeable foliar elements, principle component analyses explained 63.5% of the variation in foliar exchangeable ions across the cities (Fig 2B; PCA1, 43.8% and PCA2, 19.8%). The exchangeable ions having the strongest positive correlation with PCA1 were Al, P, K and Zn (Fig 2B). The foliar exchangeable ion with strongest loading on PCA2 was Mn (positive; Fig 2B). The patterns of both total and exchangeable foliar chemistry of red maple trees in Newark and Philadelphia forests formed two ellipses that represent the group mean for each city (Fig 2A and 2B).

### Soil bulk density, organic matter and pH

There was little variation in soil BD among forests within Newark and Philadelphia. The mean soil BD was significantly lower in Philadelphia (1.01 ± 0.04 g cm^-3^; *p =* 0.0027) compared to Newark forests (1.14 ± 0.02 g cm^-3^). However, the percent SOM was significantly higher in Philadelphia forests (13.8 ± 2.4%) compared to Newark forests (7.7% ± 0.6%; *p* = 0.016). Aqueous soil pH (1:1) was not significantly different between Philadelphia forests (4.7 ± 0.1) and Newark forests (4.5 ± 0.1; *p* = 0.11).

### Soil nutrients and stress-inducing metals

Mean concentrations of Ca, K, Mg, P, S in soils collected below red maple trees were 1.5 to 2.5-fold higher in Philadelphia forests compared to Newark forests (*p* < 0.001, Table 4). Soil Cr, Co, Ni, and Se concentrations were significantly greater in Newark forests than in Philadelphia forests (*p* < 0.001; Table 4). In contrast, soil concentrations of Al, Zn, Cu, Pb, and Cd were significantly greater in Philadelphia forests compared to Newark forests (*p* < 0.05; Table 4).

**Table 4:** Sub-canopy soil total element concentrations for the upper 0–10 cm soil horizon of 77 red maples in ten forests in Newark, DE and Philadelphia, PA.

Total soil element	Newark, DE	Philadelphia, PA	Unit
Ca	1153 ± 125^a^	**1821 ± 132**^**b**^	mg kg^-1^ DW
K	1312 ± 61^a^	**3216 ± 236.5**^**b**^	mg kg^-1^ DW
Mg	1647 ± 94^a^	**4073 ± 364.2**^**b**^	mg kg^-1^ DW
P	448 ± 35^a^	**691 ± 33**^**b**^	mg kg^-1^ DW
S	420 ± 49^a^	**638 ± 77**^**b**^	mg kg^-1^ DW
Fe	18799 ± 1634^a^	**26606 ± 1577**^**b**^	mg kg^-1^ DW
Mn	370 ± 54^n.s.^	416 ± 39^n.s.^	mg kg^-1^ DW
Zn	58 ± 6^a^	**92 ± 5**^**b**^	mg kg^-1^ DW
Cu	19 ± 2^a^	**36 ± 2**^**b**^	mg kg^-1^ DW
Al	20619 ± 1159^a^	**24006 ± 1388**^**b**^	mg kg^-1^ DW
Na	167 ± 7^a^	**184 ± 9**^**b**^	mg kg^-1^ DW
Pb	973 ± 0.6^a^	**1007 ± 91**^**b**^	μg kg^-1^ DW
Cd	1.0 ± 0.1^a^	**2 ± 0.1**^**b**^	μg kg^-1^ DW
Cr	**1165 ± 15**^**a**^	396 ± 0.4^b^	μg kg^-1^ DW
Co	**146 ± 23**^**a**^	46 ± 0.2^b^	μg kg^-1^ DW
Ni	**351 ± 74**^**a**^	120 ± 15^b^	μg kg^-1^ DW
As	**39 ± 1.5**^**a**^	35 ± 2^b^	μg kg^-1^ DW
Se	**82 ± 4**^**a**^	36 ± 2^b^	μg kg^-1^ DW

Letters ‘a’ and ‘b’ represent significant differences when α < 0.05.

Bold values represent significantly greater concentrations.

**Fig 3:**
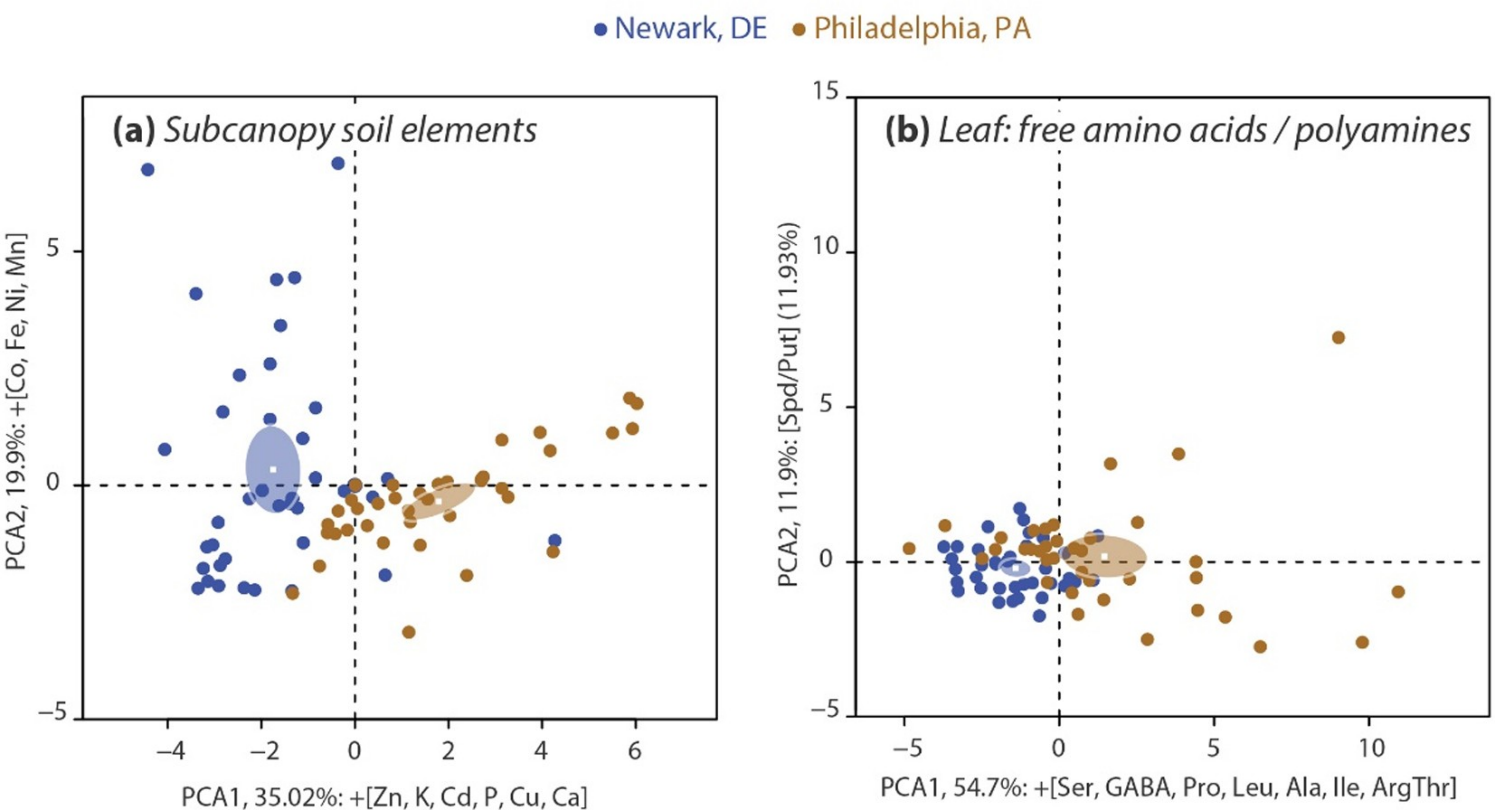
Principal component analysis for total soil elements (a) in the top 10 cm of soil and foliar free amino acids and polyamines (b) from 77 mature, native red maple trees in ten urban deciduous forests in Newark, DE and Philadelphia. PCA components that most strongly loaded on each axis are listed next to their respective axis in order of strongest to weakest. (+/-) represents values that indicate variables are positive/negative and strongly loaded on the axis. Ellipses represent group means.

Urban conditions can reveal soil elements unique to city pressures, and PCA can reveal relationships among soil elements across the two cities. PCA was used to characterize the variability in soil elements across forests in Newark and Philadelphia. The first two axes in the PCA explained 54.9% of the variation in total soil elements. The variables with the strongest positive (Zn, K, Cd, P, Cu, Ca) loadings on the first principle component (PCA1) explained 35.0% of the variation. The variables with the strongest positive (Co, Fe, Ni, Mn) loadings on PCA2 explained 19.9% of the variation in the soil chemistry (Fig 3). These strongly correlated soil variables of PCA1 and PCA2 (Fig 3A) formed two ellipses that represent the group mean of soil chemistry for Newark and Philadelphia.

### Amino acid and polyamine accumulation

There were greater concentrations of foliar free amino acids and free polyamines in the foliage of red maples in Philadelphia forests as compared to those in Newark forests. Foliar serine (Ser), Arginine + Threonine, (Arg+Thr, unseparated), proline (Pro), glutamic acid (Glu), glutamine (Gln), ornithine (Orn), and glycine (Gly) were significantly higher in red maple trees in Philadelphia forests compared to Newark forests (*p* < 0.05; Table 5). In addition, concentrations of methionine (Met) and γ-aminobutyric acid (GABA) were marginally higher in Philadelphia forests (*p* < 0.1). There were no differences in the levels of foliar Putrescine (Put) or Spermidine (Spd) between cities. Spermine (Spm) was the only free polyamine that was marginally higher in the foliage of red maples in Philadelphia (*p* = 0.08).

**Table 5:** Free amino acid and polyamine concentrations (nmol g^-1^ FW) in the foliage of 76 red maples in ten forests in Newark, DE and Philadelphia, PA.

Metabolites	Newark, DE	Philadelphia, PA	p-value
Glu	127.6 ± 6.1^a^	**151.92 ± 8.2**^**b**^	*P* = 0.020
Gln	134.2 ± 12.7^a^	**185.28 ± 9.7**^**b**^	*P* = 0.002
Arg-Thr§	46.8 ± 2^a^	**122.4 ± 5.5**^**b**^	*P <* 0.001
Gly	28.9 ± 1.5^a^	**35.4 ± 1.9**^**b**^	*P* = 0.008
Pro	37.3 ± 2.39^a^	**45.86 ± 3.5**^**b**^	*P* = 0.046
GABA	339.8 ± 15.4	**405.21 ± 31**	*P* = 0.059
Orn	0.3 ± 0.33^a^	**6.95 ± 0.6**^**b**^	*P* < 0.001
Ser	112.8 ± 7.3^a^	**204.6 ± 12**^**b**^	*P <* 0.001
Met	147 ± 30	**214.7 ± 38.6**	*P* = 0.090
Put	24.5 ± 22^n.s.^	30.52 ± 3.7^n.s.^	*P >* 0.050
Spd	25.3 ± 1.76^n.s.^	29.73 ± 2.6^n.s.^	*P >* 0.050
Spd/Put	1.2 ± 0.1^n.s.^	1.42 ± 0.2^n.s.^	*P >* 0.050
Spm	16.2 ± 0.86	**19 ± 1.4**	*P* = 0.080

Amino acids: Glu, Glutamic acid; Gln, Glutamine; Arg, Arginine; Thr, Threonine; Gly, Glycine; Pro, Proline; GABA, *gamma* Amino Butyric Acid; Orn, Ornithine; Ser, Serine; Met, Methionine; Put, Putrescine; Spd, Spermidine; Spm, Spermine.

Letters ‘a’ and ‘b’ represent significant difference in means when α = 0.05.

Bold values represent significantly greater concentrations.

^n.s.^ denotes ‘not significant.’

§Hplc system incapable of separation of these amino acids.

PCA was used to characterize the relationship of foliar metabolites (free polyamines and amino acids) in red maple trees between Newark and Philadelphia forests (Fig 3B). The first two principle components explained 66.7% of the variation in amino acids and polyamines. PCA1 and PCA2 explained 54.7% and 11.9% of the variation, respectively. Metabolites with the strongest correlation on PCA1 were Ser, GABA, Pro, Leu, Ala, Ile, and Arg+Thr (in order of strength), and all of the variables had significant positive loadings on PCA1 (Fig 3B). The variable with the strongest correlation on PCA2 was the Spd/Put ratio, which had a significantly positive loading. The two ellipses represent the group means of foliar free polyamines and amino acids in red maple growing in Newark and Philadelphia forests (Fig 3B).

### Amino acid and polyamine correlations with foliar and soil nutrients

Foliar amino acids and polyamines had significant correlations with foliar and soil nutrients and metals. Red maple trees had significant positive correlations between foliar metabolites (i.e., Ser, GABA, Pro, Leu, Ala, and Arg+Thr) and foliar B, S, and Pb (p < 0.05; S1 Table), and trees in Philadelphia had greater concentrations of these foliar metabolites and elements (Tables 2 and 5 and Figs 2A and 3b). Alternatively, red maples had significant negative correlations between foliar metabolites and foliar Mg, Mn, Cr, Co, Ni, and As (p < 0.05; S1 Table), and trees in Newark had lower concentrations of the foliar metabolites yet greater concentrations of the foliar elements (Tables 2 and 5 and Figs 2A and 3B). Similar trends were observed between foliar metabolites and soil nutrients and metals. Red maples had significant positive correlations between foliar metabolites (i.e., Gln, Arg+Thr, Orn, and Ser) and soil Ca, K, P, Mg, Zn, and Cu (p < 0.05, S2 Table), and red maples in Philadelphia forests had greater concentrations in these foliar metabolites and soil elements (Tables 4 and 5 and Fig 3A and 3B). Red maples had significant negative correlations between foliar metabolites and soil Cr, Co, Ni, and Se (p < 0.05, S2 Table), and trees in Newark had lower foliar metabolite concentrations yet greater soil Cr, Co, Ni, and Se concentrations (Tables 4 and 5 and Fig 3A and 3B).

## Discussion

### Foliar physio-biochemistry of red maple trees in Philadelphia forests significantly differed from Newark forests

Our findings supported our expectation that red maples growing in Philadelphia forests compared to those in Newark forests would demonstrate stress mitigating-acclimating responses. We found that red maples in Philadelphia forests had higher pigment, free amino acids, nutrient, and metal concentrations than red maple trees growing in Newark forests. Soils in Philadelphia were biogeochemically different and contained greater levels of magnesium which caused an imbalance in the calcium: magnesium ratio and subsequently plant uptake of calcium, magnesium and manganese. Red maple trees growing in Newark forests had higher concentrations of foliar total Mg, stress-inducing Mn, Cr, Co, and Ni, and exchangeable Mg and Mn most likely due to surface-geology-related (Cr, Co, Ni, Se) soils disturbed by previous land use (i.e., agricultural practices). Increased total chlorophyll concentration (and chlorophyll-*a*) and nutrients (N, P, K) in red maples in Philadelphia suggests a net adaptive effect on plastids (chloroplasts, mitochondria) in response to a simultaneous increase in soil nutrient availability (Ca, Mg, K, P, S, Fe, Zn, Cu; positive effects) and elevated stress-inducing metals (Na, Al, Mn, Cd, Pb; negative effects). Elevated amino acids and marginally higher spermidine in red maple trees in Philadelphia forests further suggests a possible protective, inter/intracellular signaling, and antioxidative role of the N-containing and N-storage secondary metabolites that support chloroplasts ability to biosynthesize more chlorophyll from elevated N_r_ inputs to these forests.

Overall, these findings suggest that it is the magnitude of urban intensity that determines the relative impact on foliar chemistry (chlorophyll, amino acids, polyamines, macro- and micro-nutrients, stress-inducing elements), soil physical properties (SOM, pH, BD), and the interaction of between soil and trees in Newark and Philadelphia area forests. The accumulation of amino acids, spermine, carotenoids and nutrients were all likely responsible for the detoxification of reactive oxygen/nitrogen species (ROS/RNS), ion homeostasis, and osmotic adjustment that are all important factors in physiological acclimation in red maples in Philadelphia forests [31,38,46,47,61–63]. Similar to findings of Vallano and Sparks [10] on a study of δ^15^N patterns in the foliage of red maples along a N deposition gradient, we found enriched foliar δ^15^N in red maple trees growing in Philadelphia relative to Newark. According to previously published literature, more enriched foliar δ^15^N and elevated foliar %N suggest differences in atmospheric N_r_ deposition and N sources, differences in soil N cycling rates and associated ^14^N losses, and/or within plant N allocation differences in forests between the two cities [8,14,24,26,27,61,64,65]. Elevated chlorophyll and marginally higher carotenoids observed in red maple trees in nutrient-rich soils in Philadelphia forests relative to Newark forests were strongly correlated with an accumulation of Pro, Gln, Arg, Gly, spermine, and nutrients (Ca, K, P). This is consistent with a response to excess N availability as previously reported by Minocha et al. [27,28] for chronic N addition studies conducted at the Harvard Forest, MA and Bear Brook Watershed, ME. Lahr et al. [61] reported that wildtype red maples had higher water-use efficiency (WUE), stomatal conductance and photosynthetic rates as air temperature increased in urban settings; the extent of this increase was attributed to their genetic background and local adaptation.

In the present study, red maple trees growing in urban environments with urban-influenced biogeochemistry, heavy metal-contaminated, low pH soils are likely to have responded to combined salt and heavy metal (osmotic and oxidative) stresses by triggering the accumulation of arginine, ornithine, and proline [42,44,63,66,67]. Multiple cumulative stresses (low soil pH, high N input, drought, high temperature) also associated with an accumulation of polyamines and amino acids [32,36,41]. Furthermore, these trees may have exploited novel epigenetic strategies (new genotypes) to respond to different environments that are known to regulate cell physiology [68]. In response to differing environmental gradients, gene up-regulation can result in the biosynthesis and accumulation of soluble proteins and sugars, increased ions, and increased enzymes activity (Rubisco, nitrate reductase, arginase, ornithine aminotransferase), leading to osmotic adjustment using a network of free C and N interactions to achieve ROS/RNS homeostasis [32,36,69–72]. Several studies have reported adaptive stress response traits in other plant species that were similar to those observed in red maple trees in Newark and to a greater degree in Philadelphia forests [22,73–75]. Previous reports have shown an accumulation of cellular K^+^ with salt stress [76], cellular Zn^2+^ with altered Na^+^ and K^+^ homeostasis [72], free amino acids with heavy metals exposure [33,35,77], and spermine under drought, heavy metal, and temperature stress [63,78,79]. Cellular free polyamines, amino acids, and nutrient ions likely play a significant role in cellular oxidative stress protection, signaling, and antioxidation for physiological acclimation in low pH, altered nutrient biogeochemistry, and heavy metal contaminated soils.

The accumulation of foliar chlorophyll, nitrogen, nutrients, stress metals, amino acids and spermidine in Philadelphia forests (Tables 2, 3 and 5) is evidence of a possible shift in C and N metabolic pathways via interconversions and biosynthesis of various metabolites in response to elevated N (e.g., N-containing Arg, Orn, Gln, Glu), stress-inducing metals (e.g., Pro, Ser and Gly signaling, phytochelatins (not analyzed in the present study), temperature (e.g., spermine, thermospermine (not analyzed in the present study)), and salt (e.g., spermine, K, P, Zn). The cumulative metro-scale (elevated UHI, N deposition and CO_2_) impacts and the resulting coordinated physio-metabolic shifts suggest enhanced productivity and physiological stress acclimation in Philadelphia red maples, which was observed to a lesser extent in Newark (Table 5 and Fig 3). Here, we have demonstrated that in the larger (higher population density), warmer (UHI and dryer soils), city (metro-scale impacts) with nutrient-rich soil (higher inputs of Mg, Ca, P, and K and N-S deposition) the urban conditions appear to be stimulating chlorophyll biosynthesis and inducing stress mitigating and/or acclimation responses for increased growth rate in red maples [1–3,21]. Our research provides evidence that nutrient-rich urban environments [64,72,76,80] may compensate for environmental perturbations on forest trees growing in a large city.

### Urban soil-tree interactions indicate differing above- and below-ground impacts in Newark and Philadelphia forests

We hypothesized that higher concentrations of foliar polyamines, some amino acids and nutrients in trees in Philadelphia forests relative to Newark forests would indicate more tolerance of stress of a greater magnitude of urban impacts from a large city. Forest trees in cities are subjected to multiple simultaneous above- and below-ground pressures that influence physiology, biochemistry, and stress mitigation-acclimation patterns that are unique to soil type, local environmental conditions, and metro-scale impacts [3,23,45,64]. Soil-tree interactions possibly initiate a network of molecular cross-talk with other signaling compounds within cells to allow plants to persist under stressful conditions and effectively respond to growth-modulating conditions [33,36,45,62,69,80,81]. Sub-canopy soil and foliar elements showed evidence of traffic-related sources (Zn, Cu, Pb) in Philadelphia forests as supported by red maple leaf enriched δ^15^N signature [65], and land use/land cover legacy-associated sources (Cr, Co, Se) in Newark forests, and geology-related origin (Al and Mn) in both Newark and Philadelphia forests [16,65,82–85]. Concentrations of elements reported in these forest floor soils were variable yet comparable to concentrations reported for soil in other metropolitan regions (i.e., New York, NY, Baltimore, MD, or Louisville, KY [16,85,86]).

An increase in soil acidity results in increased mobilization of Al^3+^ that can lead to plant toxicity [28,86,87]. In acidic soils with low Ca and SOM, mobile Al^3+^ competes with Ca in plant root hair channels for uptake [88]. However, Philadelphia forests had significantly greater soil Ca, P, K, Mg, S (Table 4) and SOM than Newark forests (Table 3) suggesting that plant uptake of Al, likely as AlPO_4_, was not detrimental to red maple trees in Philadelphia forests [31]. Higher amounts of SOM observed in Philadelphia forest soils may be due to more recalcitrant leaf litter containing higher levels of stress acclimating compounds (spermine and several amino acids) observed in trees (Table 5) [89] and/or these systems may have achieved steady-states where SOM decay rates would be similar to litter production rates. Higher amounts of soil N has also been linked to increased litter accumulation [90]. Alternatively, lower SOM in Newark forests might have been due to previous land use practices such as intensive agriculture that has long-term negative impacts on SOC, N and BD [90]. According to Scharenbroch et al. [91], urban landscapes are altered differently over time. These authors also suggested that the impact of site disturbance decreased rapidly over time, therefore an older city (e.g., Philadelphia, PA in the present study) is closer to a steady-state condition relative to a younger urban landscape (e.g., Newark, DE).

Soils in Philadelphia forests had similar pH (4.7) to Newark forest soil (pH = 4.5). However, the pH of soil in both of these urban forests is reflective of northeastern temperate forests that have suffered from many decades of acidic deposition and is lower than the average natural soil pH of 5.6 [92,93]. Soil acidification is reported to be exacerbated under elevated N inputs (N saturation) which may lead to an increase in leaching of base elements and possibly an increase in the solubility and plant uptake of soil Al [92,94,95]. In addition, plants may be challenged with other nutrient imbalances and possible metal toxicity (altered soil biogeochemistry: Mn, Al) in acidic soils making them more vulnerable to multiple abiotic and biotic stresses [35,56,86,92,93]. The elemental interactions between soils and trees stemming from altered edaphic properties in urban environments are likely to dictate urban tree physiology and stress mitigating/acclimation strategies at the city scale. As compared to Philadelphia, the red maples sampled in Newark forests were exposed to lower overall temperature, higher soil acidity and soil moisture from summer rainfall, all of which can increase the bioavailability of Mn^2+^ [93,96,97] which was found to be higher in red maple trees in Newark (Tables 2 and 3).

Cellular sequestration of Mn and Mg in red maple suggests Mn and Mg were possibly co-linked during accumulation [35]. Significantly higher concentrations of total and exchangeable foliar Mn and Mg were observed in red maples in Newark forests relative to Philadelphia forests although soil concentrations of Mg, P and Fe were much higher in Philadelphia. Gransee and Führs [98] reported that an increase in soil Mg reduces Mn toxicity not only by reducing Mn uptake (cation antagonism) but also by increasing the tolerance to Mn in plant tissues. Similarly, greater soil Fe concentrations have been shown to ameliorate Mn toxicity through reduced Mn uptake and translocation to leaf tissues. The reported higher foliar concentration of Mn in red maples in Newark forests may be driven in part by lower concentrations of soil Mg, P and Fe that would otherwise reduce or ameliorate foliar Mn effects compared to Philadelphia forests. Lower ratios of soil Ca/Mg (0.4) and Mn/Fe (0.16) due to different biogeochemistry in Philadelphia than in Newark forests likely influence lower plant Mn uptake and translocation to tissues (Tables 2 and 3) reducing red maples Mn sensitivity compared to more sensitive red maples in Newark forests.

The findings further suggest that the red maple trees growing in Philadelphia and Newark forests were subjected to similar anthropogenic (Zn, Cu, Pb; [84]) and surface geology-related (Al, Mn; [85]) sources of stress-inducing metals. However, the differences in the magnitude of the urban intensity between the two cities influenced the soil biogeochemistry and leaf-soil interactions differently [99]. This along with additional disparities such as reactive nitrogen deposition and air temperatures in the intensity of above-ground stressors appear to have resulted in very different foliar metabolic responses in red maples in forests in Philadelphia and Newark. The differences in total values of foliar and soil elements, foliar exchangeable ions, metabolites, soil element ratios, leaf/soil element relationships, SOM, and BD between Newark and Philadelphia forests suggest very complex but dominant metro-scale and site-specific impacts on soil-tree interactions and tree health. These findings help discern potential drivers of tree physiology and acclimation and may be useful in understanding the importance of balanced nutrient supply for plant productivity and oxidative stress mitigation for the growth and survival of urban trees.

### Red maple trees in Philadelphia forests demonstrate physiological acclimation to urban conditions

Co-occurring metro-scale and site-specific impacts influenced soil factors and plant traits differently between Newark and Philadelphia forests resulting in distinct city-specific patterns of alterations in physiology and levels of acclimation in red maple trees in the forests of each city. Similar to previously published reports on other plant species, foliar free polyamines and amino acids, and specific nutrients appear to be important players in stress-signaling, stress-mitigating, and N storage in red maple experiencing high soil N, heavy/toxic metals, salt, drought, acidity and heat stresses [27,36,42,61–63,72,76,79,93,98]. Higher levels of metal-chelating amino acids (proline, glycine), N-storing arginine and glutamine, and intermediate levels of ornithine, the driving force behind changes in N metabolites, likely accumulated in response to greater levels of cellular ROS and RNS caused by elevated heavy/toxic metals, salt ions, and N [32,33,36,67,100]. In addition, the accumulation of spermine (Table 5) with only an insignificant increase in putrescine and spermidine was likely a response to broad spectrum stresses such as salt, heat, N, and drought [36,62,63,67,78,98]. Greater metro-scale impacts of stress-inducing metals in soil (e.g., Zn, Al, Se, Cr), altered soil biogeochemistry (e.g., Ca:Mg), UHI, elevated N inputs and their combined effects in the larger city (Philadelphia) possibly result in more tightly coupled root-leaf communication. Significant positive correlations observed between several metabolites and foliar as well as soil elements in larger Philadelphia city forests but negative ones in case of smaller Newark city forests indicate not only a tight coupling between these metabolites and elemental concentrations but also different mechanisms functioning in the two ecosystems (S1 and S2 Tables). This root-leaf connection appeared to have caused higher levels of accumulation of amino acids and polyamines that are needed for physiological acclimation relative to trees in Newark forests. These metabolites are known to play an important role in cell functions like signaling, ROS homeostasis, heavy metal chelation and storage of excess N for osmotic adjustment[36,101–103].

The dynamic shift in cellular metabolites and nutrients as heavy/toxic metals and N accumulated in red maple leaves in Philadelphia forests suggests a unique shift in the C and N metabolic pathways under stressful conditions that was at least partially, if not fully, regulated by cellular nutrient concentrations [36,69,72]. It can be hypothesized that such shifts in C and N reallocation may possibly require the enzyme ornithine aminotransferase for the increased biosynthesis of arginine and subsequent production of more proline and spermine from the arginine metabolic pathway using arginase [42,44,66]. Higher foliar free amino acids and spermidine further suggests tight physiologically regulated ROS/RNS homeostasis that may be responsible for a higher level of physiological acclimation in trees in Philadelphia forests that experience multiple cumulative abiotic/biotic stress complexities relative to Newark forests.

The present study demonstrates evidence of physiological acclimation in red maple trees in Philadelphia forests, which experience a greater magnitude of urban intensity. Higher accumulations of chlorophyll, free amino acids and spermine, along with higher foliar nutrients and specific stress-inducing metals suggest higher levels of stress tolerance and higher cellular physiological acclimation in Philadelphia forests. Urban intensity can be a metro-scale phenomenon that appears to override normal above- and below-ground conditions in these forests as reflected by the altered biogeochemistry of soil, soil physical properties, soil-tree elemental interrelationships, and tree physio-biochemical patterns as demonstrated in Philadelphia and Newark forests. Greater metro-scale impacts of below-ground conditions in forests in Philadelphia seem to be a dominant influence causing potential acclimation in red maple trees.

## Conclusions

Our study shows that a greater magnitude of urban intensity was associated with particular differences in leaf physiology, biochemistry and elemental composition of red maples in urban forests of Philadelphia, which may be indicative of environmental acclimation and prolonged tree health. In Philadelphia, higher concentrations of photosynthetic pigments, N-containing metabolites, and nutrients demonstrate an acclimation response to increased nutrient loads, toxic metals, UHI, increased atmospheric CO_2_, and higher N deposition inputs. These results suggest that city size can be used as a proxy for evaluating different magnitudes of urban intensity (metro-scale impacts) on the physiology and acclimation of trees in urban forests. The observed acclimation of red maple trees to altered air and soil quality [104], while maintaining higher productivity [22], supports the suitability of this tree species for biomonitoring urban forest health and urban conditions.

## Supporting information

S1 Table Foliar metabolite correlations with foliar nutrients. Pearson correlation coefficients and p-values between foliar metabolites and foliar nutrients.
(DOCX)

S2 Table Foliar metabolite correlations with soil nutrients. Pearson correlation coefficients and p-values between foliar metabolites and soil nutrients.
(DOCX)

## References

[1] Falxa-Raymond N, Palmer MI, McPhearson T, Griffin KL. Foliar nitrogen characteristics of four tree species planted in New York City forest restoration sites. Urban Ecosystems. 2014;17:807–24.

[2] Pretzsch H, Biber P, Uhl E, Dahlhausen J, Schütze G, Perkins D, et al Climate change accelerates growth of urban trees in metropolises worldwide. Scientific Reports. 2017;7:15403 10.1038/s41598-017-14831-w 29133790 PMC5684322

[3] Gregg JW, Jones CG, Dawson TE. Urbanization effects on tree growth in the vicinity of New York City. Nature. 2003;424:183–7. Epub 2003/07/11. 10.1038/nature01728 12853954

[4] Argüeso D, Evans JP, Pitman AJ, Di Luca A. Effects of city expansion on heat stress under climate change conditions. PLoS One. 2015;10:e0117066 10.1371/journal.pone.0117066 25668390 PMC4323111

[5] Calfapietra C, Peñuelas J, Niinemets Ü. Urban plant physiology: adaptation-mitigation strategies under permanent stress. Trends in Plant Science. 2015;20:72–5. 10.1016/j.tplants.2014.11.001 25476199

[6] Farrell C, Szota C, Arndt SK. Urban plantings: ‘Living laboratories’ for climate change response. Trends in Plant Science. 2015;20:597–9. 10.1016/j.tplants.2015.08.006 26440428

[7] Grimm NB, Faeth SH, Golubiewski NE, Redman CL, Wu J, Bai X, et al Global Change and the Ecology of Cities. Science. 2008;319:756–60. 10.1126/science.1150195 18258902

[8] Craine JM, Elmore AJ, Aidar MP, Bustamante M, Dawson TE, Hobbie EA, et al Global patterns of foliar nitrogen isotopes and their relationships with climate, mycorrhizal fungi, foliar nutrient concentrations, and nitrogen availability. New Phytologist. 2009;183:980–92. Epub 2009/07/01. 10.1111/j.1469-8137.2009.02917.x 19563444

[9] Nadelhoffer KJ, Emmett BA, Gundersen P, Kjonaas OJ, Koopmans CJ, Schleppi P, et al Nitrogen deposition makes a minor contribution to carbon sequestration in temperate forests. Nature. 1999;398:145–8.

[10] Vallano DM, Sparks JP. Foliar δ15N is affected by foliar nitrogen uptake, soil nitrogen, and mycorrhizae along a nitrogen deposition gradient. Oecologia. 2013;172:47–58. Epub 2012/10/17. 10.1007/s00442-012-2489-3 23070141

[11] Stitt M, Krapp A. The interaction between elevated carbon dioxide and nitrogen nutrition: the physiological and molecular background. Plant, Cell & Environment. 1999;22:583–621.

[12] Ziska LH, Bunce JA, Goins EW. Characterization of an urban-rural CO_2_/temperature gradient and associated changes in initial plant productivity during secondary succession. Oecologia. 2004;139:454–8. Epub 2004/03/17. 10.1007/s00442-004-1526-2 15021982

[13] Foundra D, Santamouris M. Synergies between Urban Heat Island and Heat Waves in Athens (Greece), during an extremely hot summer (2012). Scientific Reports. 2017;10973:1–11. 10.1038/s41598-017-11407-6 PMC5591211 28887502

[14] Moser A, Uhl E, Rötzer T, Biber P, Dahlhausen J, Lefer B, et al Effects of climate and the urban heat island effect on urban tree growth in Houston. Open Journal of Forestry. 2017;7:428–45.

[15] Manta DS, Angelone M, Bellanca A, Neri R, Sprovieri M. Heavy metals in urban soils: a case study from the city of Palermo (Sicily), Italy. Science of The Total Environment. 2002;300:229–43. 10.1016/s0048-9697(02)00273-5 12685485

[16] Pouyat RV, McDonnell MJ. Heavy metal accumulations in forest soils along an urban- rural gradient in Southeastern New York, USA. Water, Air, and Soil Pollution. 1991;57:797–807.

[17] Livesley SJ, McPherson GM, Calfapietra C. The Urban Forest and Ecosystem Services: Impacts on Urban Water, Heat, and Pollution Cycles at the Tree, Street, and City Scale. Journal of Environmental Quality. 2016;45:119–24. Epub 2016/02/02. 10.2134/jeq2015.11.0567 26828167

[18] Krämer U. Metal hyperaccumulation in plants. Annual Review of Plant Biology. 2010;61:517–34. 10.1146/annurev-arplant-042809-112156 20192749

[19] Norton BA, Coutts AM, Livesley SJ, Harris RJ, Hunter AM, Williams NSG. Planning for cooler cities: A framework to prioritize green infrastructure to mitigate high temperatures in urban landscapes. Landscape and Urban Planning. 2014;134:127–38.

[20] Rossini M, Panigada C, Meroni M, Colombo R. Assessment of oak forest condition based on leaf biochemical variables and chlorophyll fluorescence. Tree Physiology. 2006;26:1487–96. 10.1093/treephys/26.11.1487 16877333

[21] Sonti NF, Hallett RA, Griffin KL, Sullivan JH. White oak and red maple tree ring analysis reveals enhanced productivity in urban forest patches. Forest Ecology and Management. 2019;453:117626.

[22] Bréda N, Huc R, Granier A, Dreyer E. Temperate forest trees and stands under severe drought: a review of ecophysiological responses, adaptation processes and long-term consequences. Annals of Forest Science. 2006;63:625–44.

[23] Cregg BM, Dix ME. Tree moisure stress and insect damage in urban areas in relation to heat island effects. Journal of Arboriculture. 2001;27:8–17.

[24] Silva LCR, Gómez-Guerrero A, Doane TA, Horwath WR. Isotopic and nutritional evidence for species- and site-specific responses to N deposition and elevated CO2 in temperate forests. Journal of Geophysical Research: Biogeosciences. 2015;120:1110–23.

[25] Zhang R, Wu J, Li Q, Hänninen H, Peng C, Yao H, et al Nitrogen deposition enhances photosynthesis in moso bamboo but increases susceptibility to other stress factors. Frontiers in Plant Science. 2017;8 10.3389/fpls.2017.00008 29201036 PMC5696719

[26] Minocha R, Long S, Turlapati SA, Fernandez I. Dynamic species-specific metabolic changes in the trees exposed to chronic N+S additions at the Bear Brook Watershed in Maine, USA. Annals of Forest Science. 2019;76:25.

[27] Minocha R, Turlapati SA, Long S, McDowell WH, Minocha SC. Long-Term Trends of Changes in Pine and Oak Foliar Nitrogen Metabolism in Response to Chronic Nitrogen Amendments at the Harvard Forest, MA. Tree Physiology. 2015;35:894–909. 10.1093/treephys/tpv044 26116927

[28] Bignal KL, Ashmore MR, Headley AD, Stewart K, Weigert K. Ecological impacts of air pollution from road transport on local vegetation. Applied Geochemistry. 2007;22:1265–71.

[29] McDonnell MJ, Pickett STA, Groffman P, Bohlen P, Pouyat RV, Zipperer WC, et al Ecosystem processes along an urban-to-rural gradient. Urban Ecosystems. 1997;1:21–36.

[30] Kolb KJ, Evans RD. Implications of leaf nitrogen recycling on the nitrogen isotope composition of deciduous plant tissues. New Phytologist. 2002;156:57–64.

[31] Lichtenthaler HK, Ač A, Marek MV, Kalina J, Urban O. Differences in pigment composition, photosynthetic rates and chlorophyll fluorescence images of sun and shade leaves of four tree species. Plant Physiology and Biochemistry. 2007;45:577–88. 10.1016/j.plaphy.2007.04.006 17587589

[32] Minocha R, Shortle WC, Lawrence GB, David MB, Minocha SC. Relationships among foliar chemistry, foliar polyamines, and soil chemistry in red spruce trees growing across the northeastern United States. Plant and Soil. 1997;191:109–22.

[33] Sharma SS, Dietz KJ. The significance of amino acids and amino acid-derived molecules in plant responses and adaptation to heavy metal stress. Journal of Experimental Botany. 2006;57:711–26. Epub 2006/02/14. 10.1093/jxb/erj073 16473893

[34] Wargo PM, Minocha R, Wong BL, Long RP, Horsley SB, Hall TJ. Measuring changes in stress and vitality indicators in limed sugar maple on the Allegheny Plateau in north-central Pennsylvania. Canadian Journal of Forest Research. 2002;32:629–41.

[35] Fernando DR, Marshall AT, Lynch JP. Foliar nutrient distribution patterns in sympatric maple species reflect contrasting sensitivity to excess manganese. PLoS One. 2016;11:e0157702 10.1371/journal.pone.0157702 27391424 PMC4938512

[36] Minocha R, Majumdar R, Minocha SC. Polyamines and abiotic stress in plants: A complex relationship. Frontiers in Plant Science. 2014;5: 10.3389/fpls.2014.00175 24847338 PMC4017135

[37] Näsholm Ann-Brittedfast T, Ericsson A, Nordén L-G. Accumulation of amino acids in some boreal forest plants in response to increased nitrogen availability. New Phytologist. 1994;126:137–43.

[38] Tubby KV, Webber JF. Pests and diseases threatening urban trees under a changing climate. Forestry: An International Journal of Forest Research. 2010;83:451–9.

[39] Molnár VÉ, Tóthmérész B, Szabó S, Simon E. Urban tree leaves’ chlorophyll-a content as a proxy of urbanization. Air Quality, Atmosphere and Health. 2018;11:665–71.

[40] Hoffman M, Samish RM. Free amine content in fruit tree organs as an indicator of the nutritional status with respect to potassium. Advances in Plant Nutrition. 1971;1:189–206.

[41] Moschou PN, Wu J, Cona A, Tavladoraki P, Angelini R, Roubelakis-Angelakis KA. The polyamines and their catabolic products are significant players in the turnover of nitrogenous molecules in plants. Journal of Experimental Botany. 2012;63:5003–15. 10.1093/jxb/ers202 22936828

[42] Winter G, Todd CD, Trovato M, Forlani G, Funck D. Physiological implications of arginine metabolism in plants. Frontiers in Plant Science. 2015;6:534-. 10.3389/fpls.2015.00534 26284079 PMC4520006

[43] Sharma P, Jha AB, Dubey RS, Pessarakli M. Reactive Oxygen Species, Oxidative Damage, and Antioxidative Defense Mechanism in Plants under Stressful Conditions. Journal of Botany. 2012;2012:26.

[44] Shi H, Ye T, Chen F, Cheng Z, Wang Y, Yang P, et al Manipulation of arginase expression modulates abiotic stress tolerance in Arabidopsis: effect on arginine metabolism and ROS accumulation. Journal of Experimental Botany. 2013;64:1367–79. 10.1093/jxb/ers400 23378380 PMC3598423

[45] Gregg JW, Jones CG, Dawson TE. Physiological and developmental effects of O3 on cottonwood growth in urban and rural sites. Ecological Applications. 2006;16:2368–81. 10.1890/1051-0761(2006)016[2368:padeoo]2.0.co;2 17205911

[46] Groffman PM, Pouyat RV, Cadenesso ML, Zipperer WC, Szlavecz K, Yesilonis ID, et al Land use context and natural soil controls on plant community composition and soil nitrogen and carbon dynamics in urban and rural forests. Forest Ecology and Management. 2006;236:177–92.

[47] Kaye JP, Groffman PM, Grimm NB, Baker LA, Pouyat RV. A distinct urban biogeochemistry? Trends in Ecology & Evolution. 2006;21:192–9. 16701085 10.1016/j.tree.2005.12.006

[48] McDonnell MJ, Pickett STA. Ecosystem structure and function along urban-rural gradients: An unexploited opportunity for ecology. Ecology. 1990;71:1232–7.

[49] Niinemets Ü. Responses of forest trees to single and multiple environmental stresses from seedlings to mature plants: Past stress history, stress interactions, tolerance and acclimation. Forest ecology and management. 2010;v 260:pp. 1623-39-2010.

[50] The United States Census Bureau. American fact finder. Avaialble at https://www.census.gov/ [Internet]. US Government. 2010.

[51] Ladin ZS, D'Amico V, Jaisi DP, Shriver WG. Is brood parasitism related to host nestling diet and nutrition? The Auk. 2015;132:717–34.

[52] Porra RJ, Thompson WA, Kriedemann PE. Determination of accurate extinction coefficients and simultaneous equations for assaying chlorophylls *a* and *b* extracted with four different solvents: verification of the concentration of chlorophyll standards by atomic absorption spectroscopy. Biochimica et Biophysica Acta. 1989;975:384–94.

[53] Minocha R, Martinez G, Lyons B, Long S. Development of a standardized methodology for the quantification of total chlorophyll and carotenoids from foliage of hardwood and conifer tree species. Canadian Journal of Forest Research. 2009;39:849–61.

[54] Minocha R, Shortle WC, Long SL, Minocha SC. A rapid and reliable procedure for extraction of cellular polyamines and inorganic ions from plant tissues. Journal of Plant Growth Regulation. 1994;13:187–93.

[55] Minocha R, Long S. Simultaneous separation and quantitation of amino acids and polyamines of forest tree tissues and cell cultures within a single high-performance liquid chromatography run using dansyl derivatization. Journal of Chromatography A. 2004;1035:63–73. 10.1016/j.chroma.2004.02.026 15117075

[56] Minocha R, Long S, Thangavel P, Minocha SC, Eagar C, Driscoll CT. Elevation dependent sensitivity of northern hardwoods to Ca addition at Hubbard Brook Experimental Forest, NH USA. Forest Ecology and Management. 2010;260:2115–25.

[57] Miller RO. Microwave digestion of plant tissue in a closed vessel In: Kalra Y, editor. Handbook of Reference Methods for Plant Analysis. Boca Raton, FL: CRC Press; 1998 p. 69–73.

[58] Wolf A, Beegle D. Recommended Soil Tests for Macro and Micronutrients In: A. W, editor. Recommended Soil Testing Procedures for the Northeastern United States 3rd Edition. Northeast Regional Bulletin #493: Agricultural Experiment Station, University of Delaware, Newark, DE; 2011 p. 39–48.

[59] Schulte EE. Recommended Soil Organic Matter Tests In: Sims Thomas J. WA, editor. Recommended Soil Testing Procedures for the Northeastern United States. Northeast Regional Bulletin #493: Agricultural Experiment Station, University of Delaware, Newark, DE; 1995 p. 47–56.

[60] Eckert D, Sims JT. Recommended soil pH and lime requirement tests In: Sims J. Thomas WA, editor. Recommended Soil Testing Procedures for the Northeastern United States. Northeast Regional Bulletin #493: Agricultural Experiment Station, University of Delaware, Newark, DE; 1995 p. 11–6.

[61] Lahr EC, Dunn RR, Frank SD. Variation in photosynthesis and stomatal conductance among red maple (Acer rubrum) urban planted cultivars and wildtype trees in the southeastern United States. PLoS One. 2018;13:e0197866 Epub 2018/05/26. 10.1371/journal.pone.0197866 29795659 PMC5967720

[62] Gupta K, Sengupta A, Chakraborty M, Gupta B. Hydrogen peroxide and polyamines act as double edged swords in plant abiotic stress responses. Frontiers in Plant Science. 2016;7:1343 10.3389/fpls.2016.01343 27672389 PMC5018498

[63] Nahar K, Hasanuzzaman M, Suzuki T, Fujita M. Polyamines-induced aluminum tolerance in mung bean: A study on antioxidant defense and methylglyoxal detoxification systems. Ecotoxicology. 2017;26:58–73. 10.1007/s10646-016-1740-9 27819117

[64] Kanwal S, Baig S, Hashmi I. Carbon storage and allocation pattern in plant biomass under drought stress and nitrogen supply in *Eucalyptus Camaldulensis* and *Populus Deltoides*. Pakistan Journal of Botany. 2019;51:1605–14.

[65] Redling K, Elliott E, Bain D, Sherwell J. Highway contributions to reactive nitrogen deposition: tracing the fate of vehicular NOx using stable isotopes and plant biomonitors. Biogeochemistry. 2013;116:261–74.

[66] Anwar A, She M, Wang K, Riaz B, Ye X. Biological roles of ornithine aminotransferase (OAT) in plant stress tolerance: Present progress and future perspectives. International Journal of Molecular Sciences. 2018;19. Epub 2018/11/25. 10.3390/ijms19113681 PMC6274847 30469329

[67] Gzik A. Accumulation of proline and pattern of α-amino acids in sugar beet plants in response to osmotic, water and salt stress. Environmental and Experimental Botany. 1996;36:29–38.

[68] Gömöry D, Hrivnák M, Krajmerová D, Longauer R. Epigenetic memory effects in forest trees: a victory of “Michurinian biology”? Central Eurpean Forestry Journal. 2017;63:173–9.

[69] Majumdar R, Barchi B, Turlapati S, Gagne M, Minocha R, Long S, et al Glutamate, ornithine, arginine, proline and polyamine metabolic interactions: The pathway is regulated at the post-transcriptional level. Frontiers in Plant Science. 2016;7 10.3389/fpls.2016.00007 26909083 PMC4754450

[70] Perez-Clemente RM, Vives V, Zandalinas SI, Lopez-Climent MF, Munoz V, Gomez-Cadenas A. Biotechnological approaches to study plant responses to stress. BioMed Research International. 2013;2013:654120 Epub 2013/03/20. 10.1155/2013/654120 23509757 PMC3591138

[71] Villar-Salvador P, Uscola M, Jacobs DF. The role of stored carbohydrates and nitrogen in the growth and stress tolerance of planted forest trees. New Forests. 2015;46:813–39.

[72] Zang D, Li H, Xu H, Zhang W, Zhang Y, Shi X, et al An Arabidopsis zinc finger protein increases abiotic stress tolerance by regulating sodium and potassium homeostasis, reactive oxygen species scavenging and osmotic potential. Frontiers in Plant Science. 2016;7:1272-. 10.3389/fpls.2016.01272 27605931 PMC4995212

[73] Dahlhausen J, Rötzer T, Biber P, Uhl E, Pretzsch H. Urban climate modifies tree growth in Berlin. International Journal of Biometeorology. 2018;62:795–808. 10.1007/s00484-017-1481-3 29218447

[74] Lambrecht SC, Mahieu S, Cheptou P-O. Natural selection on plant physiological traits in an urban environment. Acta Oecologica. 2016;77:67–74.

[75] Jia W, Zhao S, Liu S. Vegetation growth enhancement in urban environments of the Conterminous United States. Glob Change Biology. 2018;24:4084–94. 10.1111/gcb.14317 29777620

[76] Wang M, Zheng Q, Shen Q, Guo S. The critical role of potassium in plant stress response. International Journal of Molecular Science. 2013;14:7370–90. Epub 2013/04/04. 10.3390/ijms14047370 PMC3645691 23549270

[77] Thangavel P, Long S, Minocha R. Changes in phytochelatins and their biosynthetic intermediates in red spruce (Picea rubens Sarg.) cell suspension cultures under cadmium and zinc stress. Plant Cell, Tissue and Organ Culture. 2007;88:201–16.

[78] Fu X-Z, Xing F, Wang N-Q, Peng L-Z, Chun C-P, Cao L, et al Exogenous spermine pretreatment confers tolerance to combined high-temperature and drought stress in vitro in trifoliate orange seedlings via modulation of antioxidative capacity and expression of stress-related genes. Biotechnology & Biotechnological Equipment. 2014;28:192–8. Epub 07/08. 26019505 10.1080/13102818.2014.909152 PMC4433876

[79] Takahashi T, Kakehi J. Polyamines: ubiquitous polycations with unique roles in growth and stress responses. Annals of Botany. 2010;105:1–6. 10.1093/aob/mcp259 19828463 PMC2794062

[80] Lovett GM, Weathers KC, Sobczak WV. Nitrogen saturation and retention in forested watersheds of the Catskill mountains, New York. Ecological Applications. 2000;10:73–84.

[81] Stein RJ, Horeth S, de Melo JR, Syllwasschy L, Lee G, Garbin ML, et al Relationships between soil and leaf mineral composition are element-specific, environment-dependent and geographically structured in the emerging model Arabidopsis halleri. New Phytologist. 2017;213:1274–86. Epub 2016/10/14. 10.1111/nph.14219 27735064 PMC5248639

[82] Fraterrigo JM, Turner MG, Pearson SM, Dixon P. Effects of past land use on spatial heterogeneity of soil nutrients in southern Appalachian forests. Ecological Monographs. 2005;75:215–30.

[83] Richardson JB. Manganese and Mn/Ca ratios in soil and vegetation in forests across the northeastern US: Insights on spatial Mn enrichment. Science of The Total Environment. 2017;581–582:612–20. 10.1016/j.scitotenv.2016.12.170 28057342

[84] Trammell TLE, Schneid BP, Carreiro MM. Forest Soils Adjacent to Urban Interstates: Soil Physical and Chemical Properties, Heavy Metals, Disturbance Legacies and Relationships with Woody Vegetation. Urban Ecosystems. 2011;14:525–52.

[85] Pouyat RV, Yesilonis ID, Russell-Anelli J, Neerchal NK. Soil chemical and physical properties that differentiate urban land-use and cover types. Soil Science Society of America Journal. 2007;71:1010–9.

[86] Maas EV, Moore DP, Mason BJ. Influence of calcium and magnesium on manganese absorption. Plant Physiology. 1969;44:796–800. Epub 1969/06/01. 10.1104/pp.44.6.796 5799044 PMC396165

[87] Abrahamsen G. Sulphur pollution: Ca, Mg and Al in soil and soil water and possible effects on forest trees In: Ulrich B. PJ, editor. Effects of Accumulation of Air Pollutants in Forest Ecosystems: Springer, Dordrecht; 1983 p. 201–18.

[88] Meriño-Gergichevich C, Alberdi M, Ivanov AG, Reyes-Díaz M. *. Al^3+^- Ca^2+^ interaction in plants growing in acid soils: Al-phytotoxicity response to calcareous amendments. Journal of Soil Science and Plant Nutrition. 2010;10:217–43.

[89] Frey SD, Ollinger S, Nadelhoffer K, Bowden R, Brzostek E, Burton A, et al Chronic nitrogen additions suppress decomposition and sequester soil carbon in temperate forests. Biogeochemistry. 2014;121:305–16.

[90] Krishna MP, Mohan M. Litter decomposition in forest ecosystems: a review. Energy, Ecology and Environment. 2017;2:236–49.

[91] Scharenbroch BC, Lloyd JE, Johnson-Maynard JL. Distinguishing urban soils with physical, chemical, and biological properties. Pedobiologia. 2005;49:283–96.

[92] DeHayes DH, Schaberg PG, Hawley GJ, Strimbeck RG. Acid rain impacts on calcium nutrition and forest health. BioScience. 1999;49:789–800.

[93] Kogelmann WJ, Sharpe WE. Soil acidity and manganese in declining and nondeclining sugar maple stands in Pennsylvania. Journal of Environmental Quality. 2006;35:433–41. Epub 2006/02/04. 10.2134/jeq2004.0347 16455843

[94] Jacobson L, Hannapel RJ, Moore DP, Schaedle M. Influence of calcium on selectivity of ion absorption process. Plant Physiology. 1961;36:58–61. 10.1104/pp.36.1.58 16655470 PMC406089

[95] Alves LR, R. dRA, Gratao PL. Heavy metals in agricultural soils: from plants to our daily life (a review). Jaboticabal. 2016;44:346–61.

[96] Altland J. Managing manganese deficiency in nursery production of red maple extension faculty (nursery crops). North Willamette Research and Extension Center (NWREC) https://catalog.extension.oregonstate.edu/sites/catalog/files/project/pdf/em8905.pdf: Oregon State University; 2006.

[97] Lambers H, Hayes PE, Laliberte E, Oliveira RS, Turner BL. Leaf manganese accumulation and phosphorus-acquisition efficiency. Trends in Plant Science. 2015;20:83–90. Epub 2014/12/04. 10.1016/j.tplants.2014.10.007 25466977

[98] Gransee A, Führs H. Magnesium mobility in soils as a challenge for soil and plant analysis, magnesium fertilization and root uptake under adverse growth conditions. Plant and Soil. 2013;368:5–21.

[99] Murty D, Kirschbaum MUF, Mcmurtrie RE, Mcgilvray H. Does conversion of forest to agricultural land change soil carbon and nitrogen? a review of the literature. Global Change Biology. 2002;8:105–23.

[100] Palit S, Sharma A, Talukder G. Effects of cobalt on plants. The Botanical Review. 1994;60:149–81.

[101] Bown AW, Shelp BJ. Plant GABA: Not Just a Metabolite. Trends Plant Sci. 2016;21:811–3. Epub 2016/08/21. 10.1016/j.tplants.2016.08.001 27542324

[102] Handa AK, Fatima T, Mattoo AK. Polyamines: Bio-Molecules with Diverse Functions in Plant and Human Health and Disease. Frontiers in Chemistry. 2018;6. 10.3389/fchem.2018.00010 PMC5807879 29468148

[103] Seo SY, Kim YJ, Park KY. Increasing Polyamine Contents Enhances the Stress Tolerance via Reinforcement of Antioxidative Properties. Frontiers in Plant Science. 2019;10. 10.3389/fpls.2019.01331 PMC6834694 31736992

[104] Seifi HS, Shelp BJ. Spermine Differentially Refines Plant Defense Responses Against Biotic and Abiotic Stresses. Frontiers in Plant Science. 2019;10. 10.3389/fpls.2019.00117 PMC6376314 30800140

