# Supplementary material for: Red maple (Acer rubrum L.) trees demonstrate acclimation to urban conditions in deciduous forests embedded in cities
Source: PLoS One. 2020 Jul 24;15(7):e0236313. doi: 10.1371/journal.pone.0236313 (PMC7380610; doi:10.1371/journal.pone.0236313)
Supplement: S1 Table — Pearson correlation coefficients and p-values between foliar metabolites and foliar nutrients. (DOCX) [file pone.0236313.s001.docx]

**S1 Table.** Pearson correlation coefficients (R) between foliar metabolites and foliar nutrients and metals with associated p-values. Significant correlations are shown in bold.

|  | **Pearson Correlation Coefficients (R)** | | | | | | | | | | | | | | | | | |
| --- | --- | --- | --- | --- | --- | --- | --- | --- | --- | --- | --- | --- | --- | --- | --- | --- | --- | --- |
|  | **Al** | **B** | **Ca** | **Cu** | **Fe** | **K** | **Mg** | **Mn** | **Na** | **P** | **S** | **Zn** | **Cr** | **Co** | **Ni** | **As** | **Cd** | **Pb** |
| Put | **-0.30** | -0.14 | -0.18 | **-0.36** | -0.04 | **-0.26** | **-0.23** | 0.02 | 0.13 | **-0.35** | 0.20 | -0.14 | -0.18 | 0.00 | -0.08 | -0.21 | -0.10 | **0.26** |
| Spd | **-0.32** | -0.07 | -0.15 | -0.17 | -0.04 | -0.01 | -0.21 | -0.03 | **0.23** | -0.15 | 0.15 | -0.10 | 0.04 | -0.03 | 0.00 | **-0.30** | -0.11 | 0.17 |
| Spm | -0.07 | **0.35** | 0.04 | 0.20 | 0.19 | **0.23** | 0.00 | 0.10 | 0.20 | 0.08 | **0.33** | 0.07 | 0.09 | 0.02 | 0.10 | -0.17 | -0.08 | 0.10 |
| Spd.Put | 0.20 | 0.07 | 0.19 | **0.25** | 0.04 | **0.39** | 0.19 | -0.22 | 0.07 | **0.36** | -0.13 | 0.04 | **0.24** | -0.09 | 0.00 | -0.10 | 0.02 | -0.09 |
| Asp | -0.02 | 0.14 | -0.11 | -0.11 | **0.26** | 0.18 | -0.15 | 0.16 | 0.01 | -0.11 | **0.22** | 0.02 | -0.09 | 0.06 | 0.07 | **-0.21** | -0.21 | 0.21 |
| Glu | -0.11 | **0.26** | -0.09 | -0.04 | 0.09 | **0.30** | **-0.27** | -0.11 | -0.04 | 0.04 | **0.24** | 0.00 | -0.17 | -0.17 | -0.09 | **-0.38** | **-0.23** | **0.34** |
| Gln | -0.04 | -0.02 | -0.15 | -0.03 | -0.08 | **0.27** | **-0.25** | **-0.40** | -0.09 | 0.18 | -0.01 | -0.03 | -0.11 | **-0.32** | **-0.28** | **-0.30** | -0.14 | 0.03 |
| Ser | -0.05 | **0.31** | 0.02 | -0.05 | 0.09 | **0.24** | -0.15 | **-0.24** | 0.04 | 0.08 | **0.25** | 0.12 | -0.21 | -0.19 | -0.17 | **-0.33** | 0.02 | **0.44** |
| ArgThr | -0.11 | **0.27** | -0.12 | -0.06 | 0.13 | **0.38** | **-0.27** | **-0.37** | 0.02 | 0.13 | **0.32** | 0.12 | **-0.26** | **-0.25** | -0.22 | **-0.47** | -0.01 | **0.40** |
| Gly | 0.04 | **0.23** | 0.05 | -0.14 | 0.03 | 0.17 | -0.17 | **-0.25** | 0.06 | -0.04 | 0.00 | 0.03 | -0.08 | -0.21 | -0.18 | **-0.31** | -0.09 | **0.35** |
| Ala | -0.18 | **0.32** | -0.09 | -0.20 | 0.07 | -0.01 | -0.21 | 0.06 | 0.13 | -0.19 | **0.25** | 0.03 | -0.13 | -0.03 | -0.01 | **-0.25** | **-0.23** | **0.33** |
| Pro | -0.07 | **0.34** | -0.03 | -0.10 | -0.06 | 0.00 | **-0.23** | -0.14 | -0.01 | -0.02 | **0.24** | 0.12 | -0.20 | -0.19 | -0.19 | **-0.29** | -0.11 | **0.26** |
| Gaba | -0.17 | **0.32** | -0.09 | -0.06 | 0.05 | 0.15 | **-0.25** | -0.11 | -0.01 | -0.05 | **0.27** | 0.04 | -0.17 | -0.16 | -0.14 | **-0.27** | -0.21 | **0.26** |
| Ile | -0.01 | **0.43** | 0.04 | -0.11 | -0.02 | -0.01 | -0.17 | -0.13 | -0.07 | 0.07 | **0.28** | 0.14 | **-0.25** | -0.20 | -0.20 | -0.22 | -0.03 | **0.24** |
| Leu | -0.08 | **0.46** | 0.02 | -0.05 | 0.04 | 0.02 | -0.18 | -0.10 | -0.01 | 0.06 | **0.30** | 0.16 | **-0.22** | -0.12 | -0.09 | -0.20 | -0.06 | **0.29** |
| Orn | -0.04 | 0.13 | -0.12 | -0.12 | 0.07 | 0.20 | **-0.26** | **-0.47** | 0.06 | 0.10 | **0.32** | 0.08 | **-0.25** | **-0.26** | **-0.25** | **-0.44** | 0.07 | 0.18 |
|  | **P Values** | | | | | | | | | | | | | | | | | |
|  | **Al** | **B** | **Ca** | **Cu** | **Fe** | **K** | **Mg** | **Mn** | **Na** | **P** | **S** | **Zn** | **Cr** | **Co** | **Ni** | **As** | **Cd** | **Pb** |
| Put | **0.008** | 0.245 | 0.130 | **0.001** | 0.754 | **0.022** | **0.047** | 0.869 | 0.266 | **0.002** | 0.081 | 0.227 | 0.113 | 0.985 | 0.508 | 0.069 | 0.402 | **0.022** |
| Spd | **0.005** | 0.571 | 0.185 | 0.144 | 0.726 | 0.953 | 0.072 | 0.828 | **0.050** | 0.194 | 0.194 | 0.380 | 0.702 | 0.816 | 0.984 | **0.009** | 0.353 | 0.155 |
| Spm | 0.545 | **0.002** | 0.764 | 0.093 | 0.103 | **0.052** | 0.982 | 0.417 | 0.089 | 0.485 | **0.004** | 0.570 | 0.431 | 0.859 | 0.376 | 0.142 | 0.470 | 0.417 |
| Spd.Put | 0.087 | 0.534 | 0.097 | **0.031** | 0.759 | **0.001** | 0.101 | 0.061 | 0.524 | **0.002** | 0.262 | 0.753 | **0.034** | 0.455 | 0.966 | 0.396 | 0.885 | 0.448 |
| Asp | 0.837 | 0.247 | 0.367 | 0.367 | **0.024** | 0.119 | 0.210 | 0.178 | 0.915 | 0.342 | **0.053** | 0.866 | 0.423 | 0.590 | 0.545 | 0.072 | 0.065 | 0.066 |
| Glu | 0.339 | **0.023** | 0.457 | 0.724 | 0.433 | **0.008** | **0.021** | 0.360 | 0.734 | 0.757 | **0.034** | 0.967 | 0.157 | 0.149 | 0.433 | **0.001** | **0.043** | **0.003** |
| Gln | 0.734 | 0.858 | 0.208 | 0.807 | 0.470 | **0.019** | **0.033** | **0.000** | 0.444 | 0.128 | 0.965 | 0.780 | 0.348 | **0.005** | **0.015** | **0.009** | 0.240 | 0.790 |
| Ser | 0.657 | **0.006** | 0.874 | 0.660 | 0.420 | **0.036** | 0.189 | **0.036** | 0.719 | 0.505 | **0.027** | 0.314 | 0.067 | 0.106 | 0.150 | **0.004** | 0.838 | **0.000** |
| ArgThr | 0.334 | **0.021** | 0.323 | 0.590 | 0.262 | **0.001** | **0.018** | **0.001** | 0.870 | 0.276 | **0.005** | 0.324 | **0.025** | **0.031** | 0.060 | **0.000** | 0.935 | **0.000** |
| Gly | 0.713 | **0.043** | 0.686 | 0.245 | 0.772 | 0.136 | 0.142 | **0.034** | 0.592 | 0.751 | 0.969 | 0.780 | 0.507 | 0.077 | 0.115 | **0.007** | 0.444 | **0.002** |
| Ala | 0.112 | **0.005** | 0.467 | 0.089 | 0.563 | 0.948 | 0.076 | 0.630 | 0.265 | 0.105 | **0.031** | 0.796 | 0.264 | 0.818 | 0.913 | **0.034** | **0.046** | **0.004** |
| Pro | 0.548 | **0.003** | 0.788 | 0.407 | 0.627 | 0.998 | **0.049** | 0.218 | 0.958 | 0.858 | **0.038** | 0.294 | 0.081 | 0.095 | 0.108 | **0.011** | 0.364 | **0.024** |
| Gaba | 0.139 | **0.005** | 0.438 | 0.624 | 0.648 | 0.207 | **0.029** | 0.369 | 0.909 | 0.680 | **0.020** | 0.726 | 0.138 | 0.165 | 0.235 | **0.021** | 0.069 | **0.027** |
| Ile | 0.906 | **0.000** | 0.724 | 0.352 | 0.854 | 0.956 | 0.148 | 0.263 | 0.570 | 0.548 | **0.014** | 0.233 | **0.029** | 0.078 | 0.084 | 0.060 | 0.775 | **0.040** |
| Leu | 0.501 | **0.000** | 0.841 | 0.695 | 0.754 | 0.846 | 0.122 | 0.377 | 0.914 | 0.635 | **0.008** | 0.159 | **0.054** | 0.314 | 0.422 | 0.088 | 0.580 | **0.012** |
| Orn | 0.729 | 0.263 | 0.302 | 0.320 | 0.534 | 0.090 | **0.022** | **0.000** | 0.613 | 0.389 | **0.005** | 0.474 | **0.028** | **0.022** | **0.028** | **0.000** | 0.547 | 0.119 |
