# Supplementary material for: Red maple (Acer rubrum L.) trees demonstrate acclimation to urban conditions in deciduous forests embedded in cities
Source: PLoS One. 2020 Jul 24;15(7):e0236313. doi: 10.1371/journal.pone.0236313 (PMC7380610; doi:10.1371/journal.pone.0236313)
Supplement: S2 Table — Pearson correlation coefficients and p-values between foliar metabolites and soil nutrients. (DOCX) [file pone.0236313.s002.docx]

**S2 Table.** Pearson correlation coefficients (R) between foliar metabolites and soil nutrients and metals with associated p-values. Significant correlations are shown in bold.

|  | **Pearson Correlation Coefficients (R)** | | | | | | | | | | | | | | | | | |
| --- | --- | --- | --- | --- | --- | --- | --- | --- | --- | --- | --- | --- | --- | --- | --- | --- | --- | --- |
|  | **Al** | **Ca** | **Cu** | **Fe** | **K** | **Mg** | **Mn** | **Na** | **P** | **S** | **Zn** | **Cr** | **Co** | **Ni** | **As** | **Cd** | **Pb** | **Se** |
| Put | **0.26** | 0.10 | **0.36** | **0.24** | 0.22 | **0.26** | 0.18 | 0.12 | 0.06 | 0.06 | -0.01 | -0.06 | 0.12 | 0.02 | -0.16 | 0.01 | -0.04 | -0.17 |
| Spd | 0.15 | 0.15 | **0.29** | 0.19 | 0.06 | 0.16 | 0.22 | -0.06 | 0.07 | 0.19 | 0.03 | -0.07 | 0.16 | 0.04 | -0.13 | 0.17 | -0.02 | -0.10 |
| Spm | 0.01 | 0.13 | **0.23** | 0.07 | 0.02 | 0.00 | 0.18 | 0.03 | 0.13 | 0.19 | -0.01 | -0.04 | 0.06 | 0.04 | 0.06 | 0.10 | 0.07 | -0.08 |
| Spd.Put | -0.14 | 0.14 | -0.06 | -0.08 | -0.11 | -0.10 | 0.07 | **-0.26** | 0.11 | 0.12 | 0.12 | -0.13 | -0.09 | -0.09 | -0.09 | 0.21 | 0.03 | 0.00 |
| Asp | -0.11 | -0.04 | 0.14 | 0.08 | -0.01 | 0.07 | 0.08 | -0.10 | -0.02 | 0.12 | -0.02 | -0.01 | 0.07 | 0.04 | -0.01 | 0.10 | 0.14 | -0.11 |
| Glu | 0.12 | 0.13 | **0.36** | **0.22** | 0.13 | 0.18 | **0.30** | -0.16 | 0.17 | 0.13 | 0.08 | **-0.24** | -0.02 | -0.11 | -0.05 | 0.12 | 0.15 | -0.15 |
| Gln | **0.25** | **0.24** | **0.33** | **0.29** | **0.28** | **0.33** | **0.27** | -0.11 | 0.18 | -0.05 | **0.28** | **-0.31** | -0.15 | **-0.23** | -0.17 | 0.21 | -0.12 | **-0.22** |
| Ser | 0.08 | 0.20 | **0.29** | 0.12 | **0.30** | **0.23** | -0.03 | -0.07 | 0.21 | 0.17 | 0.06 | **-0.31** | **-0.25** | -0.21 | -0.15 | 0.13 | 0.15 | **-0.47** |
| ArgThr | 0.09 | **0.34** | **0.46** | 0.22 | **0.44** | **0.38** | 0.07 | 0.07 | **0.36** | **0.26** | **0.27** | **-0.42** | **-0.33** | **-0.28** | -0.16 | **0.31** | 0.17 | **-0.60** |
| Gly | -0.01 | 0.20 | 0.15 | 0.01 | 0.11 | 0.06 | -0.02 | -0.15 | 0.12 | 0.15 | 0.01 | **-0.23** | -0.19 | -0.15 | -0.15 | 0.12 | 0.17 | **-0.31** |
| Ala | -0.02 | 0.10 | 0.18 | 0.00 | 0.03 | 0.02 | -0.05 | 0.02 | 0.09 | 0.22 | -0.09 | -0.07 | -0.01 | -0.01 | -0.02 | 0.02 | 0.15 | **-0.23** |
| Pro | -0.01 | 0.09 | 0.09 | -0.05 | 0.01 | -0.02 | -0.03 | -0.11 | 0.08 | 0.11 | -0.15 | **-0.23** | -0.12 | -0.15 | -0.07 | -0.04 | 0.06 | **-0.24** |
| Gaba | -0.01 | 0.16 | 0.16 | 0.03 | 0.10 | 0.06 | -0.01 | 0.01 | 0.19 | 0.19 | -0.06 | -0.17 | -0.13 | -0.09 | -0.10 | 0.01 | 0.11 | **-0.24** |
| Ile | -0.01 | 0.11 | 0.10 | -0.03 | 0.07 | 0.00 | -0.05 | -0.08 | 0.13 | 0.09 | -0.11 | **-0.23** | -0.16 | -0.16 | -0.08 | -0.02 | 0.04 | **-0.30** |
| Leu | -0.05 | 0.10 | 0.14 | -0.07 | 0.07 | -0.01 | -0.11 | 0.01 | 0.13 | 0.17 | -0.09 | **-0.23** | -0.20 | -0.17 | -0.09 | 0.01 | 0.09 | **-0.34** |
| Orn | 0.09 | **0.39** | **0.37** | **0.23** | **0.43** | **0.41** | 0.05 | 0.02 | **0.35** | 0.16 | **0.28** | **-0.42** | **-0.34** | **-0.30** | **-0.31** | **0.34** | 0.08 | **-0.58** |
|  | **P Values** | | | | | | | | | | | | | | | | | |
|  | **Al** | **Ca** | **Cu** | **Fe** | **K** | **Mg** | **Mn** | **Na** | **P** | **S** | **Zn** | **Cr** | **Co** | **Ni** | **As** | **Cd** | **Pb** | **Se** |
| Put | **0.027** | 0.393 | **0.001** | **0.036** | 0.063 | **0.024** | 0.117 | 0.319 | 0.601 | 0.581 | 0.925 | 0.611 | 0.303 | 0.889 | 0.176 | 0.907 | 0.711 | 0.146 |
| Spd | 0.207 | 0.201 | **0.011** | 0.112 | 0.602 | 0.171 | 0.058 | 0.633 | 0.578 | 0.107 | 0.793 | 0.545 | 0.179 | 0.718 | 0.273 | 0.157 | 0.834 | 0.371 |
| Spm | 0.926 | 0.261 | **0.050** | 0.530 | 0.843 | 0.978 | 0.119 | 0.776 | 0.273 | 0.098 | 0.920 | 0.763 | 0.583 | 0.721 | 0.630 | 0.416 | 0.567 | 0.506 |
| Spd.Put | 0.244 | 0.237 | 0.594 | 0.476 | 0.339 | 0.413 | 0.553 | **0.026** | 0.362 | 0.299 | 0.315 | 0.281 | 0.421 | 0.456 | 0.434 | 0.069 | 0.830 | 0.970 |
| Asp | 0.367 | 0.704 | 0.218 | 0.508 | 0.940 | 0.565 | 0.488 | 0.380 | 0.862 | 0.323 | 0.879 | 0.918 | 0.539 | 0.723 | 0.901 | 0.409 | 0.221 | 0.370 |
| Glu | 0.318 | 0.249 | **0.001** | **0.054** | 0.265 | 0.112 | **0.009** | 0.162 | 0.142 | 0.265 | 0.487 | **0.036** | 0.865 | 0.330 | 0.673 | 0.289 | 0.201 | 0.204 |
| Gln | **0.031** | **0.037** | **0.004** | **0.011** | **0.015** | **0.004** | **0.017** | 0.349 | 0.130 | 0.698 | **0.014** | **0.007** | 0.192 | **0.045** | 0.147 | 0.068 | 0.321 | **0.058** |
| Ser | 0.514 | 0.078 | **0.012** | 0.300 | **0.009** | **0.050** | 0.788 | 0.543 | 0.073 | 0.138 | 0.593 | **0.006** | **0.028** | 0.067 | 0.202 | 0.251 | 0.211 | **0.000** |
| ArgThr | 0.444 | **0.003** | **0.000** | 0.062 | **0.000** | **0.001** | 0.549 | 0.573 | **0.001** | **0.024** | **0.018** | **0.000** | **0.004** | **0.014** | 0.176 | **0.007** | 0.137 | **0.000** |
| Gly | 0.906 | 0.090 | 0.202 | 0.935 | 0.339 | 0.634 | 0.875 | 0.185 | 0.306 | 0.212 | 0.899 | **0.051** | 0.102 | 0.194 | 0.190 | 0.316 | 0.153 | **0.006** |
| Ala | 0.854 | 0.406 | 0.119 | 0.999 | 0.785 | 0.877 | 0.678 | 0.881 | 0.467 | 0.059 | 0.423 | 0.532 | 0.912 | 0.905 | 0.870 | 0.852 | 0.209 | **0.044** |
| Pro | 0.908 | 0.465 | 0.462 | 0.665 | 0.953 | 0.838 | 0.819 | 0.355 | 0.497 | 0.337 | 0.205 | **0.045** | 0.287 | 0.192 | 0.562 | 0.726 | 0.593 | **0.038** |
| Gaba | 0.930 | 0.178 | 0.165 | 0.817 | 0.392 | 0.582 | 0.900 | 0.900 | 0.110 | 0.100 | 0.624 | 0.145 | 0.260 | 0.459 | 0.381 | 0.907 | 0.358 | **0.036** |
| Ile | 0.935 | 0.353 | 0.389 | 0.801 | 0.576 | 0.987 | 0.699 | 0.515 | 0.281 | 0.441 | 0.343 | **0.051** | 0.157 | 0.179 | 0.497 | 0.881 | 0.735 | **0.008** |
| Leu | 0.678 | 0.409 | 0.221 | 0.560 | 0.578 | 0.960 | 0.365 | 0.946 | 0.257 | 0.152 | 0.452 | **0.044** | 0.081 | 0.157 | 0.431 | 0.931 | 0.429 | **0.003** |
| Orn | 0.433 | **0.001** | **0.001** | **0.049** | **0.000** | **0.000** | 0.697 | 0.865 | **0.002** | 0.167 | **0.013** | **0.000** | **0.003** | **0.008** | **0.006** | **0.003** | 0.518 | **0.000** |
